# Neural innervation stimulates splenic TFF2 to arrest myeloid cell expansion and cancer

**DOI:** 10.1038/ncomms10517

**Published:** 2016-02-04

**Authors:** Zina Dubeykovskaya, Yiling Si, Xiaowei Chen, Daniel L. Worthley, Bernhard W. Renz, Aleksandra M. Urbanska, Yoku Hayakawa, Ting Xu, C. Benedikt Westphalen, Alexander Dubeykovskiy, Duan Chen, Richard A. Friedman, Samuel Asfaha, Karan Nagar, Yagnesh Tailor, Sureshkumar Muthupalani, James G. Fox, Jan Kitajewski, Timothy C. Wang

**Affiliations:** 1 Division of Digestive and Liver Disease, Department of Medicine and Herbert Irving Comprehensive Cancer Center, Columbia University, New York, New York 10032, USA; 2 Department of General, Visceral, Transplantation, Vascular and Thoracic Surgery, Hospital of the University of Munich, 81377 Munich, Germany; 3 Department of Cancer Research and Molecular Medicine, Norwegian University of Science and Technology, Pb 8905, N-7491 Trondheim, Norway; 4 Department of Biomedical Informatics, Irving Cancer Research Center, Columbia University, New York, New York 10032, USA; 5 Department of Biological Engineering, Division of Comparative Medicine, Massachusetts Institute of Technology, Cambridge, Massachusetts 02139, USA; 6 Department of Pathology and Cell Biology, Herbert Irving Comprehensive Cancer Center, Columbia University, New York, New York 10032, USA

## Abstract

CD11b^+^Gr-1^+^ myeloid-derived suppressor cells (MDSCs) expand in the spleen during cancer and promote progression through suppression of cytotoxic T cells. An anti-inflammatory reflex arc involving the vagus nerve and memory T cells is necessary for resolution of acute inflammation. Failure of this neural circuit could promote procarcinogenic inflammation and altered tumour immunity. Here we show that splenic TFF2, a secreted anti-inflammatory peptide, is released by vagally modulated memory T cells to suppress the expansion of MDSCs through CXCR4. Splenic denervation interrupts the anti-inflammatory neural arc, resulting in the expansion of MDSCs and colorectal cancer. Deletion of *Tff2* recapitulates splenic denervation to promote carcinogenesis. Colorectal carcinogenesis could be suppressed through transgenic overexpression of TFF2, adenoviral transfer of TFF2 or transplantation of TFF2-expressing bone marrow. TFF2 is important to the anti-inflammatory reflex arc and plays an essential role in arresting MDSC proliferation. TFF2 offers a potential approach to prevent and to treat cancer.

## Abstract (web summary)

During colorectal inflammation and cancer, myeloid cells accumulate in the spleen and suppress the host immunity response. In this study, the authors use a mouse model of colitis to demonstrate that upon vagus stimulation splenic memory T cells release TFF2, which suppresses the expansion of myeloid cells and cancer progression.

## Graphical abstract

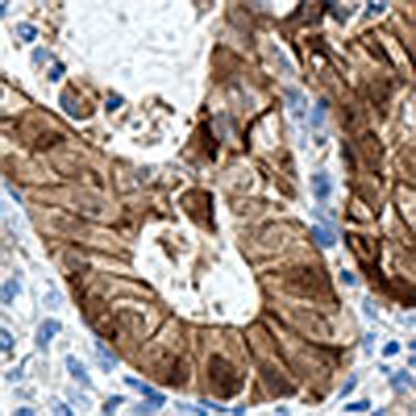

Colorectal cancer is a leading cause of cancer death^1^. Chronic inflammatory conditions, such as ulcerative colitis and Crohn's disease, increase the risk of colorectal cancer^2^. Inflammation and cancer are associated with increased myeloid CD11b^+^Gr-1^+^ cells, and these MDSCs suppress host immunity^3^,^4^.

The autonomous nervous system regulates acute inflammation through a cholinergic anti-inflammatory reflex^5^,^6^. On vagal activation, acetylcholine released from memory CD4^+^ChAT^+^ T cells binds to nicotinic receptors on macrophages, thus inhibiting cytokine production. However, the importance of the nervous system in the modulation of tumorigenesis through the anti-inflammatory reflex has not been studied.

Secreted protein trefoil factor 2 (TFF2) is produced in the stomach and duodenum where it possibly maintains mucosal integrity and restitution^7^,^8^,^9^. TFF2 mRNA has been detected also in primary and secondary lymphatic organs in rodents, but the precise cellular source and role of TFF2 in the immune compartment is not known^10^,^11^. While recombinant TFF2 attenuates colitis, the mechanisms involved have not been fully defined^12^.

In this report we show that TFF2 is expressed predominantly in splenic memory T cells, where it is regulated by the vagus nerve and suppresses colon carcinogenesis. Suppression of cancer by TFF2 was abrogated following surgical disruption of vagal innervation. Thus, we have extended the vagal efferent arc from suppression of acute inflammation to a role in coordinating procarcinogenic inflammation.

## Results

### Splenic TFF2 is regulated by vagal nerve

**Figure 1:**
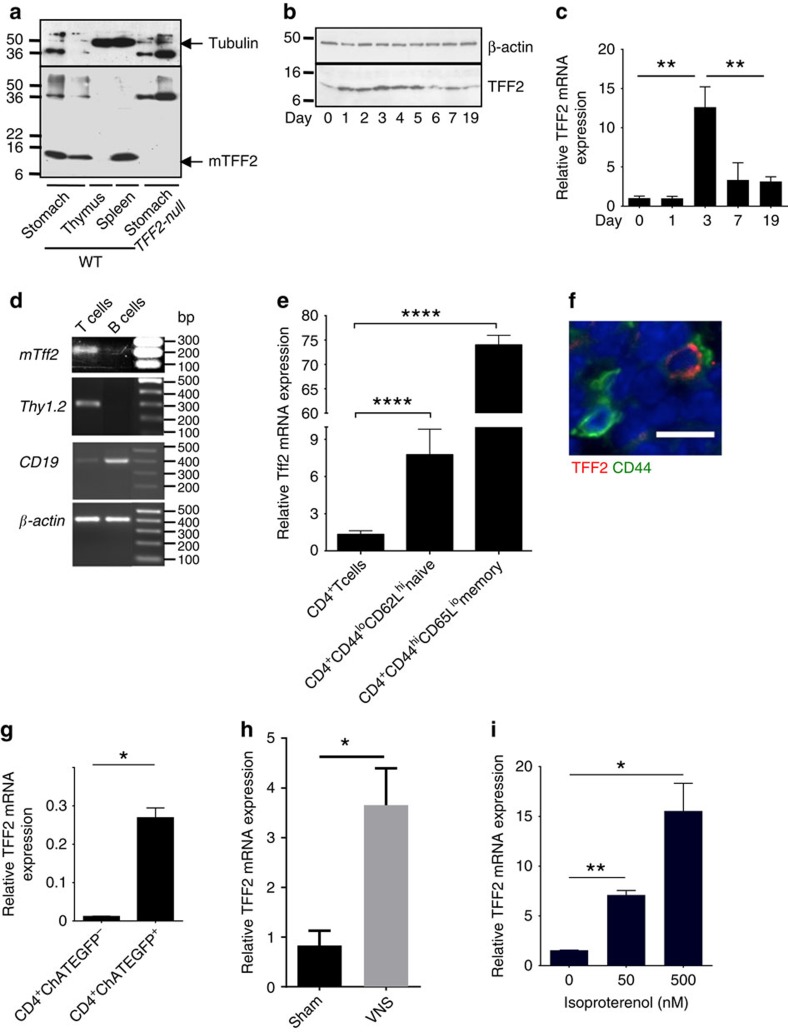
TFF2 is expressed in CD4^+^ memory T cells and regulated by the vagus nerve. (**a**) Identification of TFF2 peptide in normal spleen of wild-type mice by western blot. (**b**,**c**) Time-course change of the TFF2 protein (**b**) and mRNA (**c**) in spleen of wild-type mice after 2.5% DSS treatment. Data shown are representative of two experiments for each analysis. Data is mean±s.e.m of triplicate determinations, **P<0.01, unpaired *t*-test, two-tailed. (**d**) Detection of *Tff2* mRNA in T cells in untreated wild-type mice by semi-quantitative RT-PCR. Total mRNA from splenic T and B cells was isolated from wild-type mice and then *Tff2* mRNA was analysed along with T cells marker Thy 1.2 (second panel), B cells marker CD19 (third panel) or β-actin (quality control, bottom panel). (**e**) Expression of *Tff2* is upregulated most in splenic memory CD4^+^ T cells after DSS treatment. Unpaired *t*-test, two-tailed, the value present mean±s.e.m., **P<0.0001. (**f**) Localization of TFF2 protein in splenic CD44^+^ T memory cells (scale 10 μm). (**g**) expression of *Tff2* mRNA in ChAT^+^ T memory cells. Unpaired two-tailed *t*-test, data is mean±s.e.m., *P<0.05. (**h**) Splenic *Tff2* RNA is upregulated by vagus nerve stimulation (VNS) but not in Sham mice. Unpaired two-tailed *t*-test, data is mean±s.e.m., *P<0.05. (**i**) Isoproterenol upregulated *Tff2* mRNA expression in CD4^+^ T cells *in vitro*. Unpaired two-tailed *t*-test, data is mean±s.e.m., *P<0.05 and **P<0.01.

TFF2 protein is expressed in the spleen under normal physiological conditions (Fig. 1a). Following chemically induced colitis model (2.5% dextran sodium sulfate (DSS) *ad libitum* for 5 days), we observed a marked increase in splenic *Tff2* expression within 24–72 h that subsided over 19 days (14 days after stopping DSS, Fig. 1b,c). As TFF2 expression returned to baseline (at 19 days), splenic immature myeloid cells (IMCs) appeared to reach their peak (Supplementary Fig. 1a). Splenic *Tff2* expression is upregulated by the T-cell mitogen concanavalin A (Supplementary Fig. 1b), and we established that it was the splenic T cells, specifically, CD44^hi^CD62L^lo^ memory T cells that expressed the highest levels of TFF2 (Fig. 1d and Supplementary Fig. 1c,d), as confirmed by quantitative reverse transcriptase–PCR (qRT–PCR; Fig. 1e), immunohistochemistry (Fig. 1f) and also by *Tff2* quantitative PCR in sorted choline acetyltransferase-enhanced green fluorescent protein (ChAT-EGFP) cells (Fig. 1g). ChAT-EGFP labels an important subset of memory T cells expressing acetylcholine^6^. In addition, DSS treatment increases TFF2–EGFP-expressing memory T cells (Supplementary Fig. 1e).

Memory T cells have been implicated in the vagal efferent immunosuppressive circuit, also known as the cholinergic anti-inflammatory pathway^6^,^13^. We hypothesized that TFF2 expression in memory T cells was also regulated by the vagus nerve. Indeed, vagal nerve stimulation, mimicking the inflammatory neural circuit, resulted in the increase of splenic *Tff2* expression (Fig. 1h). Conversely, in mice with subdiaphragmatic bilateral truncal vagotomy with pyroplasty (VTPP) mice, the splenic *Tff2* response to DSS was lost (compared with control mice treated by pyloroplasty alone, Supplementary Fig. 1f). Importantly, this circuit could be partially recapitulated *in vitro* by the addition of isoproterenol (mimicking post-ganglionic vagal stimulation) to sorted splenic CD4 cells, resulting in upregulation of *Tff2* mRNA in a dose-dependent manner (Fig. 1i). Thus, the vagal pathway is important for *Tff2* expression in splenic T cells.

### TFF2 reduces colonic tumorogenesis

*Tff2*-null mice that lack both epithelial and splenic TFF2 do not differ from wild-type at a basal level^14^; however, they develop more severe colitis following DSS treatment^11^. To determine the relevance of T cell-specific TFF2 production in this acute phase response, we generated *CD2–Tff2* transgenic mice that express mouse TFF2 under a T cell-specific human CD2 promoter (Supplementary Fig. 2a–d). *CD2–Tff2* mice do not have any phenotype at baseline, with normal blood counts, body weight and life span (Supplementary Fig. 2e,f and Tables 1, 2).

**Figure 2:**
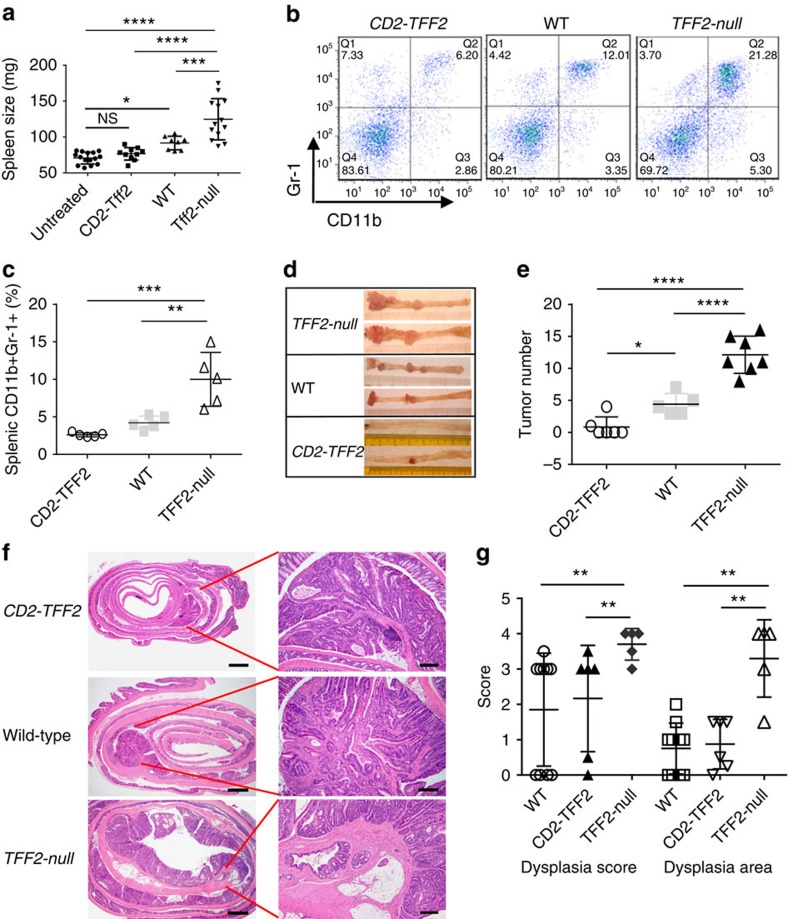
TFF2 status was associated with the expansion of IMC/MDSCs and development of colorectal cancer. (**a**,**b**) DSS treatment results in splenomegaly (**a**) and accumulation of CD11b^+^Gr-1^+^ cells (**b**) in *Tff2-null* and wild-type mice, but not in *CD2–Tff2* counterparts. Representative data from two independent experiments, 3–6 mice per group for each time point. NS, non-significant, *P<0.05, ***P<0.001, ****P<0.0001, analysis of variance (ANOVA) test following Sidak's multiple comparison test. (**c**) Highest accumulation of splenic CD11b^+^Gr-1^+^ cells in *TFF2*-null mice compare with wild-type and *CD2–Tff2* mice 5 months after AOM/DSS treatment. Data shown are representative of at least five experiments, *n*=3–6 mice in each group, Sidak's multiple comparison test after ANOVA test (**d**) Representative colons of *Tff2-null*, wild-type and *CD2–Tff2* mice 5 months after AOM/DSS treatment. (**e**) Tumour number was inversely related to *Tff2* status. Data shown are representative of at least four experiments, *n*=3–6 mice in each group, *P<0.05, ****P<0.0001, Sidak's multiple comparison test after ANOVA test (**f**) Representative hematoxylin and eosin staining of colon from *CD2–Tff2*, wild-type and *Tff2-null* mice. Scale bars are 800 μm (left panels) and 160 μm (right panels). (**g**) *Tff2-null* mice show higher dysphasia score (**P=0.008, Kruskal–Wallis test) and dysplasia area (**P<0.01, Holm–Sidak's multiple comparison test after ANOVA test), *n*=5–10 mice in group, evaluation of AOM-DSS-treated (5 months) *CD2–Tff2*, wild-type and *Tff2-null* mice.

However, on DSS treatment *CD2–Tff2* mice showed insignificant expansion of CD11b^+^Gr-1^+^ IMCs, while *Tff2-null* mice had an exaggerated splenic IMC response with splenomegaly compare with wild-type and *CD2–Tff2* mice (Fig. 2a,b and Supplementary Fig. 1a). Importantly, there were only minor differences in colonic inflammation between *Tff2-null* and wild-type controls and thus we interpreted the massive increase in IMCs in the *Tff2-null* mice to reflect an extracolonic process (Supplementary Fig. 3a–d). During chronic continuous dosing with DSS, *Tff2-null* mice died earlier than both wild-type and *CD2–TFF2* animals, but there were no differences in body weight loss during acute colitis, and little difference in cytokine levels, except for a higher colonic interleukin-1β (IL-1β) and IL-6 level in *Tff2-null* mice, with lower levels in *CD2–Tff2* mice (Supplementary Fig. 3a–d).

It has been proposed that TFF2 contributes to the cytoprotective barrier in the gut^8^. However, TFF2 was not detected at the protein level in the colonic mucosa (Supplementary Fig. 3e) and there was no difference in colonic permeability between *Tff2-null*, *CD2–Tff2* and wild-type mice in the DSS model (Supplementary Fig. 3f). Thus, TFF2 did not significantly contribute to the colonic mucosal barrier and the increased expansion of immature myeloid cells and slightly worsened colitis after DSS in *TFF2-null* mice was not due to direct colonic effects by TFF2.

The inverse relationship between TFF2 status and IMC response to injury was more striking in the azoxymethane (AOM)/DSS model of inflammatory colorectal carcinogenesis, again with a pronounced biological gradient across the TFF2 groups. Five months after AOM/DSS treatment the proportion of splenic MDSCs was lowest in the *CD2–Tff2* mice that also developed significantly fewer tumours (Fig. 2c–e and Supplementary Fig. 4a). In contrast, the *Tff2-null* mice had the most MDSCs and exhibited the greatest number of tumours, which showed a higher dysplasia grade and a greater area of dysplasia by both histology and nuclear β-catenin immunostaining (Fig. 2c–g and Supplementary Fig. 4a,b). *Tff2* mRNA was undetectable in colon of wild-type and *CD2–Tff2* mice treated with AOM/DSS; however, in contrast to decreased *Tff2* mRNA in the spleen of wild-type mice, the level of *Tff2* mRNA in the spleens of *CD2–Tff2* mice remained quite high (Supplementary Fig. 4c). Thus, given the absence of significant colonic TFF2 expression expansion of MDSC and tumour burden were inversely associated with splenic TFF2 status.

### Loss of TFF2 promotes the expansion of MDSCs

**Figure 3:**
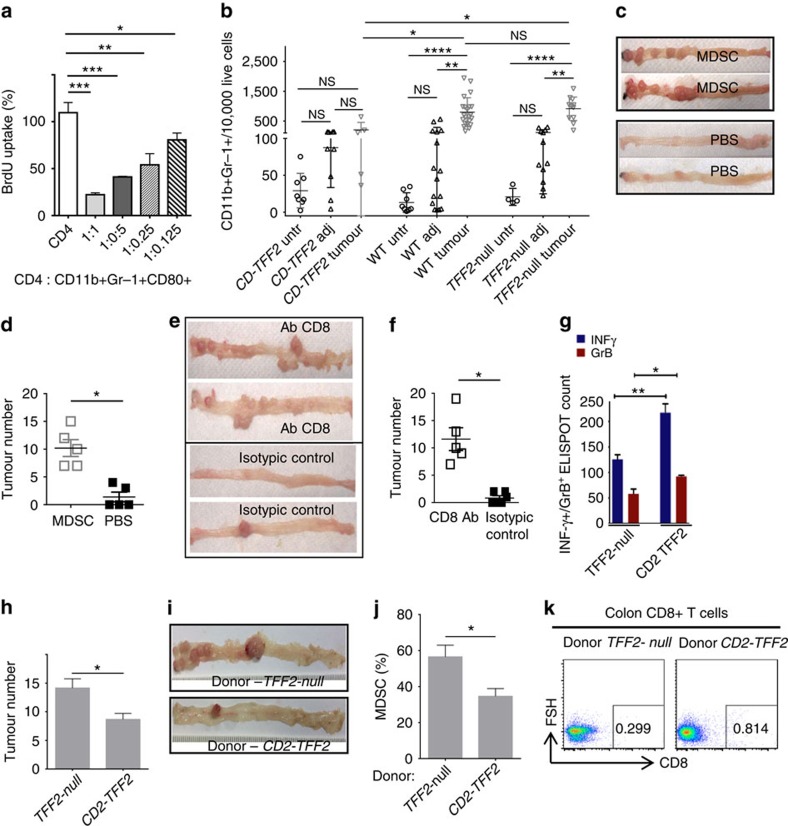
CD11b^+^Gr-1^+^ cells are MDSCs and drive tumorigenesis by suppressing CD8^+^ T cells. (**a**) Splenic CD4^+^ T cells were stimulated with anti-CD3- and CD28 plate-bounded antibody and co-cultured with sorted from *Tff2-null* mice CD11b^+^Gr-1^+^ cells in indicated ratio for 48 h. BrdU was added for the last 22 h of incubation. Values are mean±s.e.m. of three wells. Data from two experiments, *P<0.05, **P<0.01, ***P<0.001 unpaired *t*-test, two-tailed. (**b**) Accumulation of CD11b^+^Gr-1^+^ cells in adjacent (adj) and tumour tissues versus untreated colon (untr) in wild-type and *Tff2-*null mice after AOM/DSS treatment, *n*=4–6 mice in each group. ns, non significant, *P<0.05, **P<0.01, ****P<0.0001, one way ANOVA test following Sidak's multiple comparison test. (**c**,**d**) PBS (control) or MDSCs sorted from spleens and bone marrow of tumour-bearing *Tff2-null* mice were injected in *CD2–Tff2* mice treated with AOM/DSS. *N*=5 mice in each group, *P<0.05, unpaired *t*-test, two-tailed. (**e**,**f**) Depletion of CD8 T cells results in tumorigenesis in *CD2–Tff2* mice treated with AOM/DSS. (**e**) Representative pictures of the colons after CD8 antibody injections and control groups, with significant increase in cancer once CD8 cells were ablated (*P<0.05, unpaired *t*-test, two-tailed *n*=5 in each group). (**g**) Splenic CD8^+^ T cells from AOM/DSS-treated *CD2–Tff2* mice express more INF-γ and granzyme B compared with CD8^+^ T cells from AOM/DSS-treated *Tff2-null* mice. Data shown are the mean±s.e.m. of triplicates (*P< 0.05, **P< 0.01, unpaired *t*-test, two-tailed. (**h**–**k**) When transfused into wild-type mice, splenic IMCs from *Tff2-null* mice promoted tumorigenesis more than splenic IMCs from *CD2–Tff2* mice. Wild-type mice that received splenic IMCs from *Tff2-null* mice develop more tumours (**h**,**i**), show higher proportion of MDSCs (**j**) and lower colonic CD8 T cells (**k**). Error bars represent mean±s.e.m. (*P<0.05, unpaired *t*-test, two-tailed, *n*=3 mice in each group).

In the AOM/DSS model, CD11b^+^Gr-1^+^ cells in the spleen and colonic tumours were heterogeneous, but with a preponderance of Ly-6G^+^ over Ly-6C^+^ subset (Supplementary Fig, 4d–f). Splenic CD11b^+^Gr-1^+^cells from wild-type and *TFF2*-null mice showed low expression of CD40 antigen, moderate level of CD86, variable expression of CD11c, CD31, CD115 and major histocompatibility complexII, and high expression of F4/80, CD80 and PD-L1 (Supplementary Fig. 5a). Both CD80 and PD-L1 suppress anti-tumoral T-cell cytotoxicity in cancer^15^,^16^ and also could be detected on MDSC in tumour (Supplementary Fig. 5b). Splenic CD11b^+^Gr-1^+^CD80^+^ cells inhibited INF-γ production (Supplementary Fig. 5c) and the *in vitro* proliferation of CD4^+^ T cells activated with CD3/CD28 antibody (Fig. 3a). Neutralizing CD80 antibody abrogated the MDSC suppression of T-cell proliferation (Supplementary Fig. 5d). In contrast, splenic CD11b^+^Gr-1^+^ cells from *CD2–Tff2* mice expressed low levels of CD80 and did not suppress proliferation or interferon-γ (IFN-γ) expression of activated T cells (Supplementary Fig. 5a,e,f). Splenic CD11b^+^Gr-1^+^ cells from *Tff2-null* and wild-type mice express higher arginase I activity compared with those from *CD2–Tff2* mice, and lower nitric oxide (NO) activity, but generate high levels of ROS (reactive oxygen species; Supplementary Fig. 5g–i). Together, these data confirm that TFF2 from T cells suppresses the development of splenic MDSCs, which may be a critical step in modulating cancer risk.

There was a significant accumulation of CXCR4-expressing MDSCs around colonic tumours (50- to 100-fold), along with increased expression of pro-tumorigenic cytokines IL-17A and IL-1β (Fig. 3b and Supplementary Fig. 6a–d), in *Tff2-null* and wild-type mice compared with *CD2–Tff2* mice. IL-17A is a key cytokine promoting colonic tumorigenesis, stimulating and mobilizing granulocytes into sites of inflammation^17^,^18^. As expected, MDSCs from tumours generate high ROS activity (Supplementary Fig. 5i). Interestingly, the number of dendritic cells (defined as CD45^+^Ly-6G^-^F4/80^−^CD11b^+^CD11c^+^) was higher in tumours from *CD2–Tff2* mice versus tumours from the other two groups, suggesting a protective role for dendritic cells (Supplementary Fig. 6e). To prove that CD11b^+^Gr-1^+^ cells directly contributed to tumour progression, we performed adoptive transfer of MDSC sorted from spleen and bone marrow of tumour-bearing *Tff2-null* mice into AOM/DSS-treated *CD2–Tff2* mice^19^. The transfer of MDSCs from *Tff2-null* mice resulted in a marked (10-fold) stimulation of tumour growth in *CD2–Tff2* mice (Fig. 3c,d), converting the tumour-resistant *CD2–Tff2* mice into a tumour susceptible phenotype.

CD8 T cells play a key protective role in anti-tumour immune response in colorectal neoplasia^20^,^21^. Depletion of CD8^+^ T cells in *CD2–Tff2* mice treated with AOM/DSS resulted in a marked (10-fold) increase of tumour growth (Fig. 3e,f), indicating that the protective effect of Tff2 overexpression depended on CD8^+^ T-cell immunity. Splenic CD8^+^ T cells from AOM/DSS-treated *CD2–Tff2* express higher levels of IFN-γ and granzyme B (GrB) compared with splenic CD8^+^ T cells from AOM/DSS *Tff2-null* mice (Fig. 3g). There was a direct relationship in the AOM/DSS model between colonic CD8^+^ T cells and TFF2 status, with *Tff2-null* mice having no detectable colonic CD8 mRNA expression (Supplementary Fig. 6f). AOM/DSS-treated wild-type mice receiving splenic IMCs from untreated *CD2–Tff2* mice developed a lower number of tumours and a lower proportion of MDSC but a higher proportion of CD8^+^ cells in the colon compared with wild-type mice that received splenic IMC from *Tff2-null* mice (Fig. 3h–k). These data indicate a critical role for the spleen in the development of early MDSCs. Overall, the association between splenic Tff2 and cancer risk is in keeping with the well-recognized pro-tumorigenic effect of MDSCs and their suppression of tumoricidal CD8^+^ T cells^20^,^22^.

The importance of T cells as a source of TFF2, as opposed to epithelial gastric cells, was confirmed by bone marrow transplantation of *CD2–Tff2*, wild-type or *Tff2-null* donor bone marrow into wild-type mice (5–6 mice per group), followed by the DSS colitis protocol. Wild-type mice that received *CD2–Tff2* bone marrow suffered the mildest colitis, while recipients of *Tff2-null* bone marrow had slightly more severe disease (measured by weight loss, spleen size, colonic shortening and colonic IL-1β expression) (Supplementary Fig. 7a–d). More importantly, following the AOM–DSS protocol, recipients of *Tff2-null* bone marrow as expected developed the greatest tumour burden (*n*=3 per group, Supplementary Fig. 7e).

### TFF2 deficiency is responsible for the expansion of MDSCs

**Figure 4:**
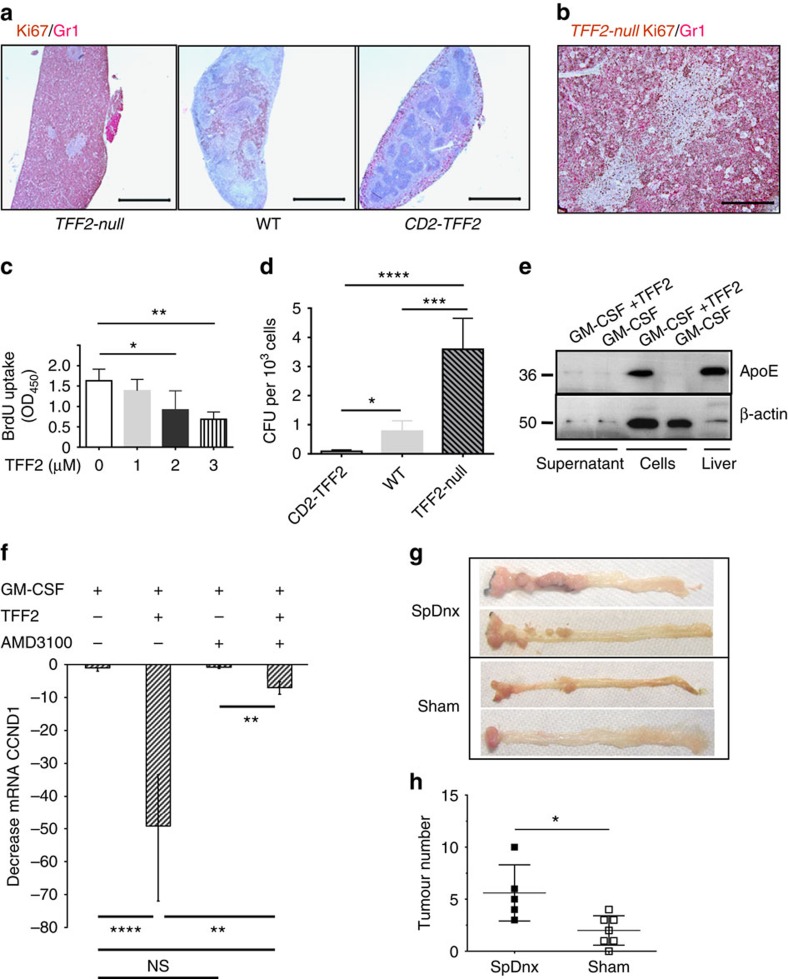
TFF2 inhibited cancer through suppression of MDSC proliferation. (**a**,**b**) Expansion of Gr-1^+^ cells in red pulp due to their high proliferation in *Tff2-null* mice treated with DSS. Ki67 (brown) and Gr-1 (red) immunostaining of spleens from DSS-challenged mice. Scale bar, 500 μm (**a**) and 100 μm (**b**). (**c**) Recombinant TFF2 suppresses proliferation of IMCs in response to TFF2 *in vitro.* Sorted CD11b^+^Gr-1^+^ cells from spleen of DSS-treated *Tff2-null* mice were GM-CSF with or without TFF2 at indicated concentrations. Proliferation was measured by BrdU uptake. Values are mean±s.e.m. of 3–4 replicates (*P<0.05 and **P<0.01), unpaired two-tailed *t*-test. (**d**) Increased CFUs from spleens of wild-type and *Tff2-null* mice treated with DSS, compared with *CD2–Tff2* mice. *n*=3–4 mice in each group, two experiments, *P<0.05, ***P<0.001, ****P<0.0001, Sidak's multiple comparison test after analysis of variance test. (**e**) rTFF2 induced expression of membrane-bound ApoE in CD11b^+^Gr-1^+^ cells. (**f**) TFF2 suppressed cyclin D1 via CXCR4 receptor. Sorted CD11b^+^Gr-1^+^ cells were cultured with GM-CSF alone, GM-CSF with rTFF2, GM-CSF with AMD3100 or GM-CSF with rTFF2/AMD3100, values are mean±s.e.m. of three replicates, unpaired two-tailed *t*-test. (**g**,**h**) Splenic denervation promotes tumour development. (**g**) Representative picture of colon from SpDnx (denervated spleen) and Sham mice. (**h**) tumour number in SpDnx and Sham mice, *n*=5–7 mice in each group, *P<0.05, unpaired *t*-test, two-tailed.

DSS treatment resulted in proliferation of CD11b^+^Gr-1^+^ cells in the spleen that was inhibited in a TFF2-dependent fashion (Fig. 4a,b and Supplementary Fig. 8a). A pulse of BrdU 3 h before sacrifice revealed that the *Tff2-null* mice had the greatest proportion of proliferating IMCs (13%) followed by wild-type (8%) and *CD2–Tff2* (1%) mice (Supplementary Fig. 8a). Recombinant TFF2 (rTFF2) directly inhibited IMC proliferation *in vitro* in a dose-dependent gradient (Fig. 4c), mediated, at least in part, by downregulation of cyclin D1 mRNA in IMCs/MDSCs (Supplementary Fig. 8b,c). TFF2 deficiency enhanced the colony forming unit potential of splenocytes grown in granulocyte-macrophage progenitor propagating media (Fig. 4d) and the proportion of splenic granulocyte-macrophage progenitors (Lin^−^IL-7Rα^−^c-kit^+^Sca-1^-^CD16/32^hi^CD34^+^) by flow cytometry (Supplementary Fig. 8d). Thus, TFF2 released from splenic T cells suppressed proliferation of early splenic myeloid progenitors.

### TFF2 suppresses myeloid cells via CXCR4 receptor

To identify likely candidates for TFF2-mediated suppression of myeloid cell expansion, we performed gene expression microarray. DSS-induced splenic CD11b^+^Gr-1^+^ cells from *Tff2-null* mice were cultured with granulocyte–macrophage colony-stimulating factor (GM-CSF) only (control) or GM-CSF plus purified mouse rTFF2 (Supplementary Fig. 8e). We compared the gene expression profiles of TFF2-treated and -untreated IMCs. 5,810 genes had a greater than twofold change in expression (Supplementary Table 3). Positive regulators of cell cycle (cyclin D1 and cyclin E1) were downregulated (eight- and twofold, accordingly), while negative regulators of cell cycle (such as Nurp1) were upregulated, in keeping with TFF2-mediated suppression of IMC proliferation (validated by qRT–PCR, Supplementary Fig. 8f and Supplementary Table 3). ApoE was one of the most upregulated genes (∼90-fold), a finding confirmed by qRT–PCR (Supplementary Fig. 8g and Supplementary Table 3). ApoE has been shown to retard metastasis^23^,^24^, and cell-surface proteoglycan-bound ApoE can directly suppress the proliferation of haematopoietic stem and progenitor cells, limiting expansion of myeloid cells^23^. Indeed, addition of rTFF2 to sorted IMCs induced the expression of the biologically relevant cell-bound ApoE (Fig. 4e). The suppression of tumour growth in response to TFF2 overexpression in the AOM/DSS model was not, however, significantly altered by knockout of the *ApoE* gene (Supplementary Fig. 8h), suggesting that there may be other important signalling targets beyond ApoE.

The CXCR4 receptor is highly expressed on MDSC from blood and colonic tumour tissues from CRC patients^25^ and TFF2 binds to and acts through the CXCR4 receptor^26^,^27^,^28^,^29^, CXCR4 expression on splenic CD11b^+^Gr-1^+^ cells was increased in DSS-treated and in tumour-bearing mice (Supplementary Fig. 8i,j). Given the close association between rTFF2 treatment of IMCs and suppression of cyclin D1, we measured the expression of cyclin D1 to test whether TFF2 suppressed proliferation of MDSC through the CXCR4 receptor. We used AMD3100 to block CXCR4 on IMCs *in vitro* (Fig. 4f). Treatment with AMD3100 reduced the TFF2-mediated downregulation of cyclin D1 (up to 80%), while AMD3100 alone had no effect on cyclin D1 level.

If TFF2 expression by splenic CD4 T cells is a component of the anti-inflammatory reflex, it should follow that physical disruption of splenic innervation would accelerate tumorigenesis in the AOM/DSS model. To specifically denervate the spleen without otherwise altering innervation of the gastrointestinal tract, we resected nerves along the splenic artery. Wild-type mice with splenic denervation (SpDnx) showed a statistically greater tumour number along with higher proportion of MDSC in spleen and bone marrow compared with control (Sham) mice receiving Sham surgery (Fig. 4g,h and Supplementary Fig. 8k–m). Thus, SpDnx in the setting of carcinogenesis leads to a marked increase in myeloid cell expansion and more rapid progression to colorectal cancer.

### *TFF2* gene delivery confers therapeutic effect on cancer

**Figure 5:**
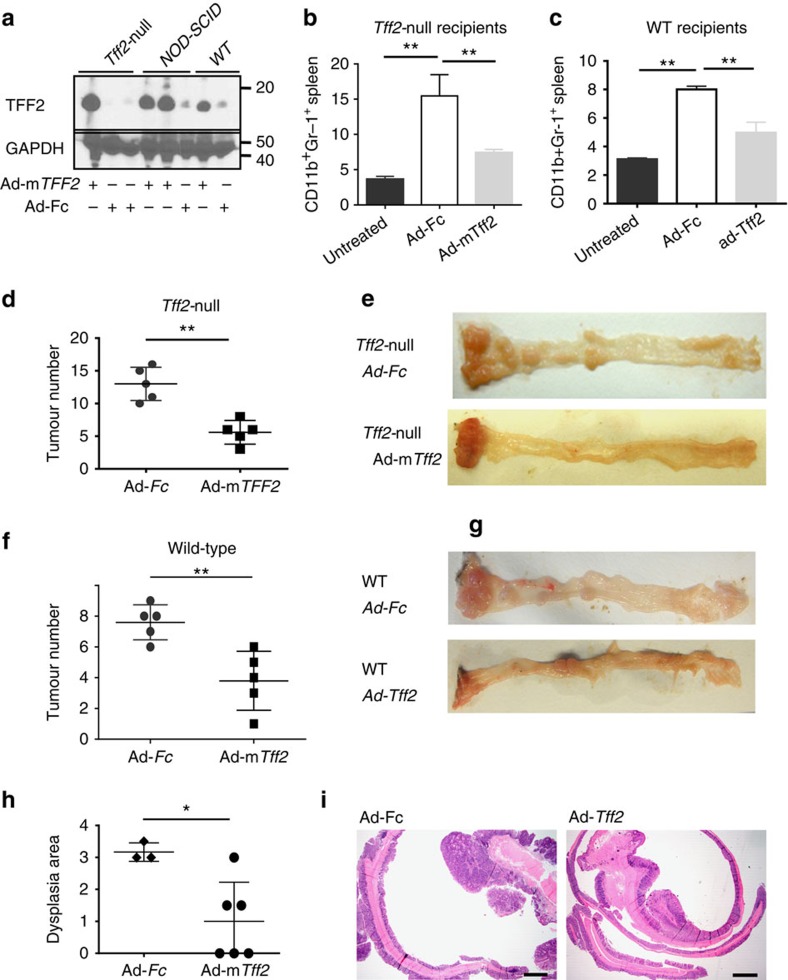
Adenoviral delivery of TFF2 reduced colorectal carcinogenesis. (**a**) Detection of recombinant TFF2 by western blot in serum from *Tff2-null*, NOD-SCID and WT mice 5 days after Ad-m*Tff2* treatment (5 × 10^8^ pfu per mouse). (**b**,**c**) Ad-*Tff2* reduced splenic CD11b^+^Gr-1^+^ cells compared to the Ad-Fc treated controls in both *Tff2-null* (**b**) and wild-type mice (**c**) following AOM/DSS protocol, **P<0.01, unpaired *t*-test, two-tailed. (**d**,**e**) *Tff2-null* and wild-type mice (**f**,**g**) treated with Ad-m*Tff2* showed lower numbers of tumours compared with control mice after AOM/DSS treatment, four experiments with *n*=4–5 mice in each group, **P<0.01, unpaired *t*-test, two-tailed or Holms-Sidak's multiple comparison test after analysis of variance test. (**h**) Significant reduction in the dysplasia area in wild-type mice, *n*=3–6 mice in each group, *P<0.05, unpaired *t*-test, two-tailed. (**i**) Hematoxylin and eosin staining of colon from wild-type mice with Ad-Fc and Ad-*Tff2* injections.

Having established splenic TFF2 as an important factor within the vagally mediated anti-inflammatory reflex arc, we wondered whether exogenous TFF2 delivery could be used to suppress MDSCs and cancer. We infected *Tff2-null* and wild-type mice with an adenovirus vector Ad-Tff2 that expressed mouse TFF2 or a control vector Ad-Fc expressing the Fc fragment of IgG (Supplementary Fig. 9a,b). After one dose of Ad-Tff2 (5 × 10^8^ pfu/mouse), TFF2 was detected in peripheral blood of *Tff2-null*, wild-type and nonobese diabetic/severe combined immunodeficiency (NOD–SCID) mice (Fig. 5a). Both wild-type and *Tff2-null* mice injected with Ad-Tff2 had fewer peripheral MDSCs in blood and spleen (Fig. 5b,c and Supplementary Fig. 9c), and developed significantly fewer tumours versus control (Fig. 5d-g). On histopathology, the area of dysplasia was significantly reduced in Ad-*Tff2* treated versus control mice (Fig. 5h,i). In contrast, there was no difference in tumour number in immunodeficient Rag2^−/−^ mice injected with Ad-TFF2 versus Ad-Fc adenovirus (Supplementary Fig. 9d), indicating once again that TFF2 suppresses tumorigenesis via the adaptive immune system.

## Discussion

Splenic TFF2 is a secreted peptide expressed predominantly in memory T cells, co-localized with ChAT-EGFP cells shown to mark memory CD4^+^ T cells^6^. TFF2 is induced *in vivo* by vagal stimulation and *in vitro* in CD4^+^ T cell by adrenergic stimulation, and induction by DSS treatment was blocked by vagotomy. Thus, splenic TFF2 is regulated by the inflammatory reflex, which in the acute setting results in the release of acetylcholine by memory CD4^+^ T cells and suppression of tumor necrosis factor-α production by macrophages^6^. Our findings suggest that the production of TFF2 by ChAT+ T cells represents a more delayed response, preventing proliferation of myeloid progenitors and the development of MDSCs following chronic stress. In contrast to the mild reduction in histologic colitis in *CD2–Tff2* transgenic mice in response to DSS treatment, we noted marked reduction in later tumour development in response to AOM/DSS. This was due to a reduction in MDSCs, since tumour suppression could be overcome by adoptive transfer of MDSCs from *TFF2-null* mice, and abrogated by immune depletion of CD8^+^ T cells.

MDSCs are increased in blood and tumour tissues from patients with advanced colorectal cancer^30^,^31^,^32^. In the setting of cancer and inflammation, the spleen becomes an important source of myeloid cells in animal models and human patients^33^,^34^. MDSCs suppress T-cell immunity through PD-L1, Arg-1, iNOS and ROS, although they also have direct pro-tumorigenic effects^25^. TFF2 acts via CXCR4 receptor that is highly expressed on MDSCs in patients with ovarian cancer, promoting their accumulation^35^, and on MDSCs in colorectal cancer^25^. Previous studies have shown that TFF2 binds to CXCR4 with low affinity^26^ and while relatively high concentrations (0.5–0.8 μM) of trefoil peptides are required for signalling^36^,^37^,^38^, the normal physiologic concentration of TFF2 may be high^39^. Further studies are needed to explore possible correlations between circulating TFF2 and the levels of MDSC. TFF2 upregulated ApoE but *ApoE−/−/CD2–Tff2* mice did not develop significantly more tumours than *CD2–Tff2* mice, suggesting some functional redundancy within this system. Overexpression of TFF2 led to a slight increase in tumour-associated dendritic cells, which might also contribute to tumour immunity. Finally, SpDnx in wild-type mice treated with AOM/DSS also resulted in both a marked increase in MDSCs and in tumour development, confirming the importance of the anti-inflammatory reflex in modulating tumour immunity.

TFF2 is lowly expressed in the intestine^10^, but no TFF2 protein could be detected in the colon of our AOM/DSS-treated mice. Furthermore, the relative sufficiency of TFF2 did not impact on intestinal barrier function. Thus, it is unlikely that local colonic production of TFF2 was responsible for the observed effects. The contribution of circulating TFF2, derived from the stomach, could not be absolutely excluded^40^. However, we observed marked differences in tumour development in mice that received wild-type versus *TFF2-null* versus *CD2–Tff2* bone marrow transplants, thus indicating the predominant role of hematopoietic, rather than epithelial, TFF2 expression at least for colorectal carcinogenesis. Finally, the marked increase in colonic tumour development following SpDnx underscores the critical role for the splenic response to carcinogenic stress. Our model, informed by earlier work, begins with the release of noradrenaline from the post-ganglionic vagus nerve in response to injury (Supplementary Fig. 10). This stimulates splenic memory T cells to upregulate TFF2 expression that acts in a paracrine fashion on local IMC/MDSCs and myeloid progenitors via the CXCR4 receptor. TFF2 suppresses IMC/MDSC proliferation in part through downregulation of cyclin D1 (and possibly through upregulation of cell-bound ApoE) thus reducing MDSCs and liberating anti-tumorigenic CD8^+^ T cells to inhibit the development of cancer. This vagally mediated circuit can be enhanced by exogenous delivery of TFF2, either through bone marrow transplantation or by an adenoviral vector, both of which suppressed MDSCs and prevented cancers. These data indicate that TFF2 is a critical component in suppressing splenic MDSCs as a part of the vagal inflammatory reflex, and suggests its potential utility in combined cancer therapy.

## Methods

### Mice

*C57/BL6* wild-type, Rag2^−/−^ , B6.129P2-*Apoe*^*tm1Unc*^/J mice were purchased from Jackson Laboratories (Bar Harbor, ME), *Tff2-null* mice were described earlier^14^, TFF2–BAC–EGFP–*Cre* transgenic mice that express an EGFP–Cre fusion protein and *CD2–Tff2* mice were generated in our laboratory, both on *C57BL/6* background, *Cxcr4*::EGFP mice (also on *C57BL/6* background) were kindly provided by Richard J. Miller (Northwestern University Medical School, USA). Sex and age matched mice were used at 6–12 weeks of age. To generate the *CD2–Tff2* mice, the mouse gene *Tff2* was cloned downstream into hCD2 promoter into *Eco*RI site of expressing vector. For this purpose site for *Eco*RI site was incorporated in primers for PCR amplification of mouse *Tff2* sequence using respective mouse complementary DNA (cDNA) library clone BC050086 (Open Biosystem, Baltimore, MD). The forward primer was 5′-ATTGAATTCGCCACCATGCGACCTCGAGATGCC-3′. The reverse primer was 5′-AATTGAATTCTCAGTAGTGACAATCTTCCACAGAC-3′. Resulting construct was transfected into *Escherichia coli* Stbl2 competent cells, clones were verified for presence and proper orientation of *Tff2* gene by sequencing using forward primer located in CD2 and reverse primer located within *TFF2* sequence. Transgenic mouse lines expressing the Tff2 protein in T cells were generated in Transgenic Core Facility of Columbia University, on a *B6CBA/F2* background. The offspring were screened for transgene integration by PCR analysis using primers selected for promoter part of *CD2* and *Tff2* gene. The offspring were tested for the presence of transgenic *CD2–Tff2* mRNA transcript spleen and thymus. Transgenic mice were backcrossed to *C57BL/6* for more than 10 generations before using in experiments. All animal protocols were in accordance to guidelines by the National Institutes of Health and approved by the Institutional Animal Care and Use Committee at Columbia University. The TFF2–BAC–EGFP–*Cre* transgenic mice were generated by BAC recombineering (clone RP23-332C16 purchased from Children's Hospital Oakland Research Institute), as previously described^41^. Recombineering primers amplified the EGFP–Cre–pA–fNf cassette with 60 bp homology arms upstream and downstream of the *TFF2* translational stop codon in exon 4. We generated three founder lines, all of which displayed a similar pattern of expression throughout various organs (stomach, pancreas and spleen). As a result, all lines were backcrossed six generations to C57BL/6 J and Line 3a used for all experiments performed here according to the guidelines of the Institute of Comparative Medicine at Columbia University.

### Induction of colitis

In the experiments involving DSS-induced colitis, 8 to 12 weeks old sex-matched *Tff2-null*, *CD2–Tff2* and wild-type mice were used unless specified otherwise. For induction of chronic colitis, *Tff2-null*, *CD2–Tff2* and wild-type mice were given 2.5% DSS (molecular weigh 36,000–50,000; MP Biomedicals, Solon, OH, USA) dissolved in drinking water provided *ad libitum* for 5 days, followed by plain water for next 14 days. Mice were analysed at day 19.

### Tumour model

Mice (age 6–8 weeks) were injected intraperitoneally (i.p.) with AOM (10 mg kg^−1^ body weight) and after 1 week 2.5% DSS water was given for 7 days. Mice were killed 5 months later. For experiments with adenovirus system, mice were injected with AOM/DSS as above, and then they were treated with one dose of Ad-m*Tff2* (5 × 10^8^ p.f.u. per mouse) every 2 weeks via tail injection and analysed 3 months later. Macroscopically visible tumours were counted and their sizes were measured. Colon sections were analysed for histopathology scoring.

### Vagus nerve stimulation

Mice were anaesthetised with ketamine (100 mg kg^−1^) and xylazine (10 mg kg^−1^) i.p. A midline abdominal incision was made and the left subdiaphragmatic vagus nerve trunk was isolated. Two bipolar platinum electrodes were applied to the nerve (Plastics One). Electrical stimulation was delivered at 1 V, 2 ms, 5 Hz for 240 min by a stimulation module (STM100A) controlled by the AcqKnowledge software (Biopac Systems). The abdomen is closed in two layers. In Sham-operated animals only an abdominal incision was made and the left subdiaphragmatic vagus nerve trunk isolated. Depth of anaesthesia was monitored by the hind paw reflex and adapted by titrating ketamine and xylazine when mice regained the reflex. Four hours after vagus nerve stimulation yohimbine (2 mg kg^−1^) was administered. Mice were euthanized 180 min later and spleen was harvested.

### Bilateral subdiaphragmatic truncal vagotomy and pyloroplasty

Mice were anaesthetised with ketamine (100 mg kg^−1^) and xylazine (10 mg kg^−1^) i.p. A midline abdominal incision was made and the vagotomy was performed by dividing and cutting both vagal trunks immediately below the diaphragm. To prevent food retention and fatal gastric dilatation a pyloroplasty was performed by cutting the pylorus lengthwise and closing it in a perpendicular fashion using two single 8–0 non-absorbable sutures. The abdominal cavity was closed in a two layer fashion. In control animals (Sham operation), the subdiaphragmatic vagal nerves were isolated but not cut and a pyloroplasty was performed.

### Splenic denervation

Three-week-old *C57BL/6* mice were injected with AOM (azoxymethan) 10 mg kg^−1^ and 3 weeks later SpDnx was performed as follows. Mice were anaesthetised using isoflurane. A midline laparotomy allowed exteriorization of the pancreatic tail after mobilizing the spleen. After dissecting the pancreas until the level of the portal vein, the coeliac artery and the branching of the splenic artery were identified. The nerve bundles were subtly removed using micro-surgical instruments under an operating microscope (magnification × 25). After careful irrigation of the entire abdominal cavity with 0.9% sterile and pre-warmed saline, the sutures was checked for cessation of bleeding and the procedure was ended by closing the abdominal muscles using 5–0 vicryl sutures (Ethicon), and the skin was stapled using 9.0-mm staples (Reflex Skin Closure Systems). A total of 0.1 mg kg^−1^ buprenorphine–HCl in normal saline was injected subcutaneously for analgesia. Mice were given 2 weeks to recover after surgery and AOM was injected twice (5 mg kg^−1^) with interval 1 week between injections. Then mice were given three cycles of 1.5% DSS for 7 days with tap water between DSS. Mice were sacrificed after 4.5–5 months.

### Permeability measurement

Mice were given 2.5% DSS water during 5 days, and then FITC-dextran 4000 was injected as follows. After anaesthesia, a midline laparotomy incision was made. A 2-cm long segment of the colon inside was created with two vascular hemoclips without disrupting the mesenteric vascular arcades. The length of the intestine between two clips was injected with 50 μl of 100 mg ml^−1^ FITC-dextran 4000 (Sigma-Aldrich) solution per mouse. Mice were sacrificed after 1 h and fluorescence was measured in the plasma with a fluorimeter. Dilutions of FITC-dextran in normal mouse serum were used as a standard curve, and absorption of 100 μl of serum or standard was measured in a fluorometer at 488 nm.

### Cell sorting

Splenic CD11b^+^Gr-1+ cells were labelled with PerCP 5.5-conjugated Gr-1 and APC-conjugated CD11b antibodies and sorted using sorter FASCAria.

### Depletion of CD8 cells *in vivo*

Four weeks after AOM/DSS treatment mice were injected i.p. with anti-mouse CD8a antibody (clone 53.6.72, BioXcell) or corresponding isotypic control (clone 2A3, BioXcell) at 12 mg kg^−1^ in sterile DPBS twice weekly for 12 weeks.

### MDSC adoptive transfer

For experiments of MDSC adoptive transfer, *CD2–Tff2* mice were injected with AOM (10 mg kg^−1^), and a week later were treated twice with 2.5% DSS during 7 days with weekly interval between two treatments. Two weeks later CD11b^+^Gr-1^+^ from spleens of *Tff2-null* tumour-bearing mice were labelled with antibody CD11b–APC and Gr-1–PerCP5.5 and sorted by using FASC Aria (BD Bioscience), and then sorted cells were intravenously injected into *CD2–Tff2* (2–3 × 10^6^ per mouse) once a week during 6 weeks. Mice were analysed 5 months after treatment with AOM.

### Adoptive transfer of IMC from *Tff2-null* mice and from *CD2–Tff2* mice

Wild-type mice (CD45.1) were treated AOM/DSS and after 13 weeks injected with CD11b^+^Gr-1^+^ cells (3 × 10^6^ per mouse) sorted from spleens of naive *Tff2-null* and *CD2–Tff2* mice. Mice were analysed 5 weeks later.

### Bone marrow transplantation

Mice were lethally irradiated with single dose 9 Gy and 6 h later were reconstituted with bone marrow cells (3.5–5 × 10^6^ per mouse) by tail vein injection. Transplanted mice were allowed to rest 4 weeks before administration of DSS (2.5%) or AOM/DSS protocols. In tumour studies mice were injected AOM and then were treated with three cycles of 2% DSS water.

### Histopathology

Formalin-preserved colons were processed and embedded in paraffin according to standard procedures. Colon and tumour sections (5 μm) were stained with hematoxylin and eosin and examined by a pathologist blinded to the experimental groups.

### NO and ROS analysis

For NO analysis 100 μl of culture supernatant were mixed with equal volume of Griess agent and nitrite concentrations were measured by comparing the absorbance values of the test sample with standard curve. Absorbance was measured at 550 nm after 15 min incubation at room temperature.

The production of ROS in MDSCs was determined with the dye 29,79-dichlorofluorescein diacetate (Sigma-Aldrich). Cells from mice treated with AOM/DSS were incubated at 37 °C in RPMI 1640 in the presence of 2.0 mM 29,79-dichlorofluorescein diacetate for 30 min, then labelled with antibody CD45–PE–Cy7, CD11b–APC, Gr-1–PerCP5.5 for 20 min. Analysis was then performed by flow cytometry.

### Immunohistochemistry

For granulocyte and Ki67 staining, formalin-fixed paraffin-embedded spleens were stained with anti-Gr-1 (Ly-6G/Ly-6C, clone RB6-8C5) antibody labelled with biotin (dilution 1:200, BioLegend), anti-mouse Ki67 antibody (dilution 1:200) and secondary antibodies (dilution 1:200, Dako).

For CD44 and TFF2 immunostaining 250 μg blefeldin A was injected intravenously into wild- type mice 6 h before sacrificed^42^. Splenic cryosections were incubated with goat anti-mouse CD44 (1:1,000, BD) and rabbit affinity-purified antibody against C-terminal part of molecule (0.01 μg ml^−1^) for 2 h. After washing with PBS-Tween 20, the sections were when incubated with Alexa Fluor 594-conjugated anti-rabbit IgG and Alexa Fluor 488-conjugated anti-goat IgG (BD) for 1 h. Subsequently, splenic cryosections were stained with 4,6-diamidino-2-phenylindole (BD) at 0.1 mg ml^−1^ and observed by fluorescence microscopy with 420 and 590 nm filters.

### FACS analysis

Cells were labelled with appropriate antibodies and sorted using sorter FASCAria (Becton Dickinson). Phycoerythrin (PE)-conjugated anti-mouse-CD11c (clone N418), -CD86 (clone GL1), -CD80 (clone 16-10A1), -CD31 (clone 390), -CD40 (clone 1c10), -major histocompatibility complexII (clone I-A), -CD115 (clone AFS98), -F4/80 (clone BM8) (from eBioscience). Anti-mouse PE–Cy7–CD8a (clone 53-6.7), Alexa Flour 700-CD4 (clone GK1.5), APC–Cy7–CD3ɛ (clone 145-2C11), PerCP–Cy 5.5–Gr-1 (Ly-6G/Ly-6C, clone RB6-8C5), APC–CD11b (clone M1/70), PE/Cy7–CD45 (clone 30-F11), FITC–Ly-6G (clone RB6-8C5) were purchased from PharMingen or Biolegend, PerCP/Cy5.5–CD16/32 (clone 93), Pacific Blue Ms CD3/Ly-6G(Ly-6C)/CD11b/CD45R(B220)/Ter-119, PE/Cy7–CD117 (clone 2B8), APC–CD115 (clone AfS98), PE–CD127 (clone A7R34), APC–Cy7–Ly6A/E (clone D7), FITC–CD34 (clone RAM34), APC–CD4 (clone RM4-5), FITC–CD44 (IM7), PerCP/Cy5.5–CD62L (MEL-14) were purchased from PharMingen, eBiocsience or Biolegend. Dead cells were discriminated using 4,6-diamidino-2-phenylindole. Samples were analysed on LSRII flow cytometer and the data files were analysed using FlowJo 5.5.5 software.

### ELISPOT assay

Sorted spleen CD8+ T cells from either *Tff2-null* or *CD2–Tff2* AOM/DSS-treated tumour-bearing mice were mixed with irradiated naive splenocytes at 1:1 ratio. Cells were then stimulated with 1 μg ml^−1^ each anti-CD3/CD28 antibodies (Biolegend) for 48 h. ELISPOT assay was performed by using Mouse IFN-γ/Granzyme B dual-color Elispot kit (R&D Systems) according to manufacturer's instructions. Plates were scanned and counted for INF-γ or Granzyme B stained dots.

### Semi-quantitative and qRT–PCR

Splenic T and B cells were isolated from wild-type mice by negative selection using immunomagnetic separation kit (Myltenyi Biotech, Inc.). To confirm the validity of separation procedure total mRNA was extracted from T- and B-cell population and subjected to semi-quantitative PCR analysis for T and B cell-specific marker Thy 1.2 and CD19, respectively. Total RNA was isolated and reverse transcribed to cDNA with Superscript III (Invitrogen, Carlsbad, USA). Selected ApoE and three cell cycle related genes (cyclin D1, cyclin E1, Nupr 1), which differential expression in TFF2 treated cells was identified by microarray hybridization analysis, were tested by the real-time qRT–PCR assay.

Mouse TFF2 primers (5′-GTCAGCTCGCAAGAATTGTG-3′, (exon 3–4) 5′-GGCAGTAGCAACTCTCAGTA-3′, probe: 5′-/56-FAM/CAGGGCACT/ZEN/TCAAAGATCAGGTTGGA/3IABkFQ/-3′) mouse CD8b1 primers: (5′-TGGCCGTCTACTTTTACTGTG-3′, (exon 5–6) 5′-GGCGCTGATCATTTGTGAAAC-3′, probe: 5′-/56-FAM/TGAAGTGAA/ZEN/TTCGGGCTCTCCTCC/3IABkFQ/-3′); mGAPDH primers: IDT Mm.PT.39a.1 (exon 2–3), TaqMan Universal PCR Master mix.

Mouse ApoE−/− primers (5′-GATCCGATCCCCTGCTCAGA-3′; 5′-TCTGTCACCTCCGGCTCTCC-3′), cyclin E1 (5′-TTCAGCCTCG GAAAATCAGACC-3′; 5′-GCACACCTCCATTAGCCAATCC-3′), Nupr1 (5′-GGTCGGACCAAGAGAGAAGCTG-3′; 5′-TGGTGTCTGTG GTCTGGCCTTA-3′), IL-1β (5′-CAACCAACAAGTGATATTCTCCATG-3′; 5′-GATCCACACTCTCCAGCTGCA-3′), IL-17A (5′-TCCAGAATGTGAAGGTCAACC-3′; 5′-TATCAGGGTCTTCATTGCGG-3′), and reference gene GAPDH (5′-ACGGACCCCAAAAGATGAAG-3′; 5′-TTCTCCACAGCCACAATGAG-3′), Mastermix (Qiagen) with SYBR Green.

For calculation of fold augmentation RNA amounts were normalized to GAPDH mRNA. Assays were performed on ABI 7500 thermal cycler (Applied Biosystem). Results were calculated using 2^−ddCt^ formula. Statistical significance of different gene expression (dCt) was evaluated using Student's *t*-test for paired samples.

When sorted CD11b^+^Gr-1^+^ cells were used total mRNA was isolated by using RNAqueus-micro Kit (Ambion, Grand Island, NY).

### CD11b^+^ Gr-1^+^ cells proliferation assay

CD11b^+^ Gr-1^+^ cell proliferation was determined using BrdU Cell Proliferation Assay Kit (Calbiochem, Germany) or mRNA cyclin D1 was quantified by qRT–PCR. Live CD11b^+^Gr-1^+^ cells from DSS-induced (day 19) or tumour-bearing mice were sorted from spleen and cultured 7 days in complete RPMI 1640 medium (Invitrogen) supplemented with 10% FCS (HyClone), 50 μM β-mercaptoethanol, 1 mM penicillin-streptomicin, GM-CSF in concentrations 2.5 ng ml^−1^. Recombinant mouse TFF2 was added in concentration as indicated. Every 48 h half of medium was replaced with fresh culture medium. Cell proliferation was determined by addition of BrdU during the final 18 h of culture accordingly protocol of manufacture. When specified, CD11b^+^ Gr-1^+^ cells were preincubated for 30 min with AMD3100 (0.6 μM final concentration) or antibody CD80 (10 μg ml^−1^) on ice, then cells were cultured as above.

### T cell proliferation assay

CD4+ T cells were sorted from untreated *Tff*2-null- mice and stimulated with 2.5 μg ml^−1^ plate-bound anti-CD3 and 2.5 μg ml^−1^ CD28 antibodies (BD Pharmigen) for 24 h. Live CD11b^+^Gr-1^+^ cells were sorted from spleen of *Tff2-null* AOM/DSS-treated mice (5–6 months after treatment) and cultured at indicated ratios with activated T cells in 96-well flat bottom plates in complete RPMI 1640 medium (Invitrogen) supplemented with 10% FBS, 10 mM HEPES, 1 mM penicillin-streptomycin and 50 μM β-mercaptoethanol. T cells proliferation was assessed after 72 h culture by pulsing with BrdU during the final 18 h of culture accordingly protocol of manufacture (Calbiochem, Germany). Supernatant was used for IFN-γ measurement by using commercial BD OptEIA Set kits (BD Biosciences, Sydney, Australia).

### Generation of adenovirus expressing m*Tff*2

Adenoviral vector (pAdlox), recombinant Ad-GFP and Ad-Fc (human Fc fragment of IgG1) were kindly provided by Dr Jan Kitajewski (Columbia University, NYC). The full-length m*Tff2* cDNA fragment was generated by PCR amplification using phCMV3-m*Tff2* construct and subcloned into pAdlox vector by In-fusion HD Cloning Kit (Clontech Co.). The construct of pAdlox-mT*ff*2 was confirmed by DNA sequencing, then, linearized by SfiI and packaged in HEK293 cells. The expression of recombinant m*Tff2* in 293 cells was verified by western blot. All of the viral particles were purified by caesium chloride density gradient centrifugation and titrated by TCID50 method.

### Microarray analysis

Splenic CD11b^+^Gr-1^+^ cell were sorted by flow cytometry from spleens of *Tff2*-null mice (*n*=4) taken on day 19 after single cycle with 3% DSS and split into two halves. One half was cultured for 7 days at density 1 × 10^6^ cells per ml in 3 ml complete RPMI 1640 medium (10% FCS, ES grade, Invitrogen) supplemented with GM-CSF (2.5 ng ml^−1^) alone while another half with an addition of 3 μM of mouse rTFF2. mRNA from eight samples was purified by using Trizol reagent (Invitrogen). Total RNA was reverse transcribed, amplified, labelled and hybridized to Mouse Genome 430A 2.0 arrays (Affymetrix) at The Core Facility of Columbia University. Data from the hybridized chips were scanned and analysed using Bioconductor and R software^43^,^44^. All eight chips passed recommended QC tests^45^. Normalization was performed using GCRMA^46^,^47^and statistical analysis was performed using Limma^43^. 5,810 transcripts which were differentially expressed with a Benjamini-Hochberg False Discovery Rate^48^, fdr<0.05 were found. The array data was deposited in the Gene Expression Omnibus, accession number GSE47596.

### Western blotting

Tissues (spleen, stomach mucosa, thymus, colon) were collected in RIPA lysis buffer supplemented with complete protease inhibitor cocktail (Roche) and homogenized. CD4^+^ T cells were isolated from spleen of wild-type mice on day 3 after treatment with 3% DSS and stimulated with cell activation cocktail (with brefeldin A) for 16 h. Samples were resolved by 18% SDS–PAGE and transferred to 0.2-μm pore-size PVDF membrane (Immobilon-Psq, Millipore). Membranes were blocked in 5% non-fat milk, incubated overnight at 4 °C with rabbit antibodies raised against C-end of mouse TFF2 molecule (1 μg ml^−1^) and probed with horseradish peroxidase-tagged secondary antibodies. Blots were developed with SuperSignal West Femto Maximum Sensitivity Substrate Kit (Pierce ECL). (Supplementary Figures 11).

### Cytokine ELISA

Elisa assay was performed with commercial BD OptEIA Set kits (BD Biosciences, Sydney, Australia).

### *In vivo* bromodeoxyuridine labelling

Mice were subjected to DSS protocol to induce colitis, and then injected with BrdU i.p. (1 mg per 20 g body weight) 3 h before killing them. Splenic cells were stained for CD11b^+^, Gr-1^+^ and intracellular BrdU staining was performed by using FITC BrdU flow kit (BD Pharmigen, San Diego) according to manufacturer's instructions.

### Statistical analysis

Graphs and analyses were calculated using GrapPadPrism. *P* values were determined by using two-tailed unpaired Student's *t*-test. For three or more groups an analysis of variance followed by Sidak's or Holm–Sidak's multiple comparison test or Kruskal–Wallis test was performed. The following designations apply to all figures: NS, non-significant, *P<0.05, **P<0.01, ***P<0.001, ****P<0.0001. *P* values<0.05 were considered significant. Statistical analysis of survival curves was performed using log-rank test.

## Additional information

**Accession codes:** The microarray data have been deposited in the Gene Expression Omnibus (GEO) under accession code GSE47596.

**How to cite this article:** Dubeykovskaya, Z. *et al.* Neural innervation stimulates splenic TFF2 to arrest myeloid cell expansion and cancer. *Nat. Commun.* 7:10517 doi: 10.1038/ncomms10517 (2016).

## Supplementary Material

Supplementary Information Supplementary Figures 1-11, Supplementary Tables 1-3
