# Supplementary material for: Neural innervation stimulates splenic TFF2 to arrest myeloid cell expansion and cancer
Source: Nat Commun. 2016 Feb 4;7:10517. doi: 10.1038/ncomms10517 (PMC4742920; doi:10.1038/ncomms10517)
Supplement: Supplementary Information — Supplementary Figures 1-11, Supplementary Tables 1-3 [file ncomms10517-s1.pdf]

Supplementary Figures

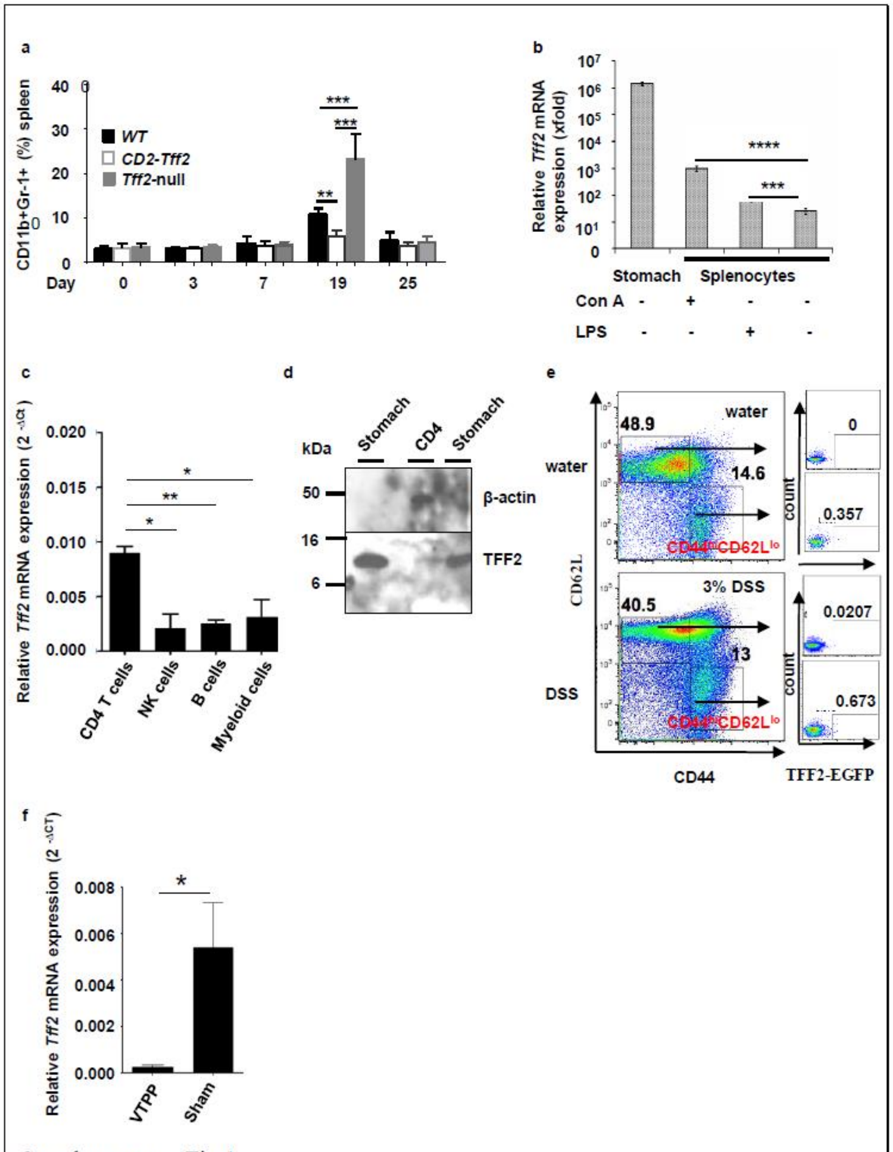

Supplementary Fig.1

Supplementary Figure 1. Expression of TFF2 in splenic T cells. (**a)** Accumulation of $CD11b^+Gr\text{-}1^+$ cells in spleen upon 2.5% DSS treatment. The values presented as mean ±s.d. per each group at chosen time point (*n*=3-7 at each time point, day 19 **P<0.01, ***P<0.001, one-way ANOVA test following Sidak's multiple comparison test) (**b**) T-cells mitogen concanavalin A (con A) induced increase of *Tff2* mRNA in splenocytes of wild-type mice. Data shown are the mean ± s.e.m. of triplicates (Student's t-test, two-tailed, **** P<0.0001, *** P<0.001). (**c**) Most *Tff2* mRNA express in $CD4^+$ T cells in normal spleen of wild-type mice. Data are shown is mean ± s.e.m. of triplicate determinations, t-test, two-tailed, *P<0.05, **P<0.01. (**d**) Western blot analysis of TFF2 expression in splenic $CD4^+$ T cells. CD4 T cells were isolated from DSS-treated mice (day 3) and stimulated with cell stimulation cocktail with bleferdin A. (**e**) TFF2 expression increases followed DSS treatment in naïve ($CD44^{lo}CD69L^{hi}$) and memory splenic $CD4^+$ ($CD44^{hi}CD69L^{lo}$) T cells. TFF2-BAC-EGFP-*Cre* transgenic mice were given 3% DSS for 7 days and then splenocytes were analyzed for expression of EGFP by flow cytometry. (**f**) Splenic TFF2 response is lost in vagotomized (VTPP) compare with Sham mice. N=4 mice in each group, the values presented are the mean ± s.e.m., t-test, two-tailed, *P<0.05)

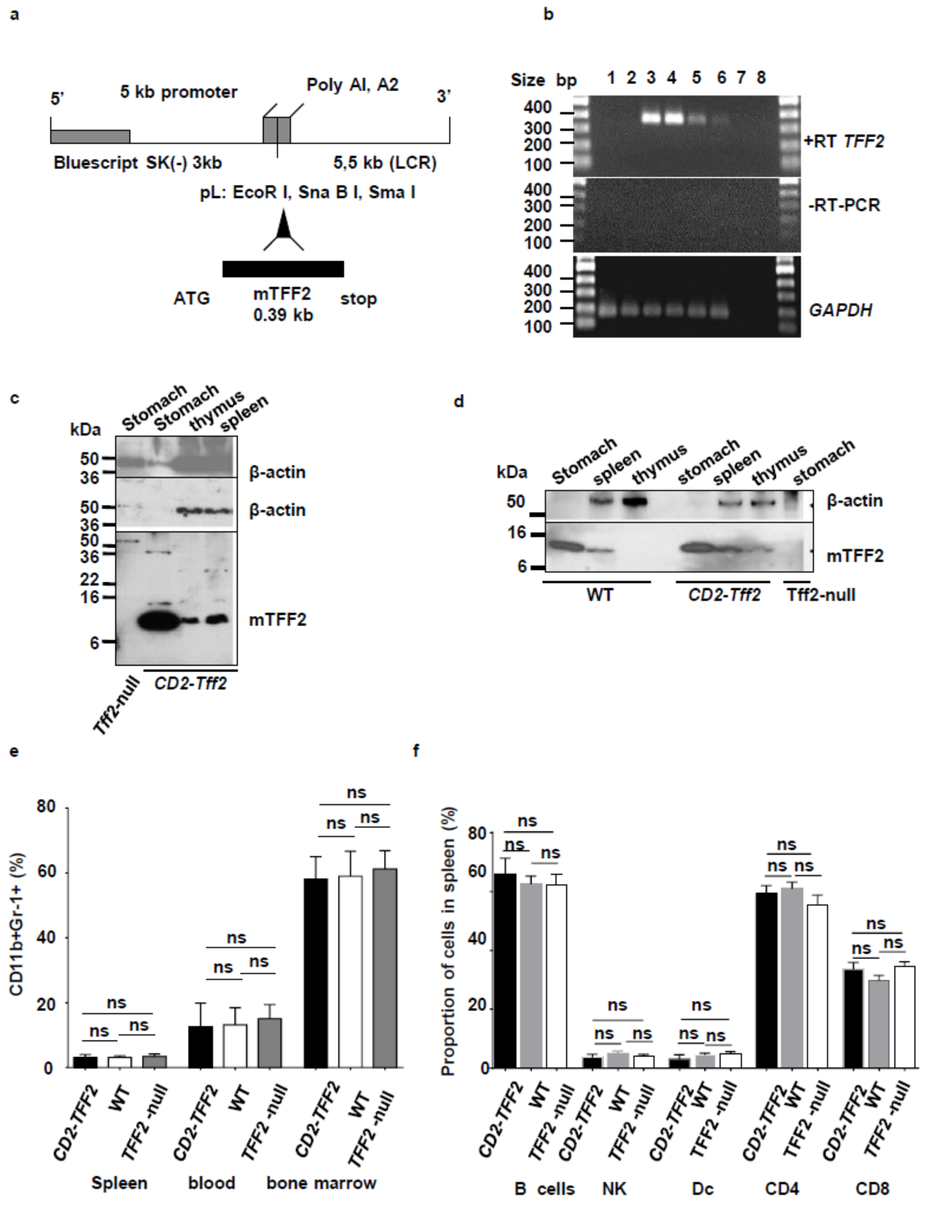

Supplementary Fig.2

Supplementary Figure 2. Characterization of *CD2-Tff2* mice. (**a**) A scheme of *Tff2* gene cloning into hCD2 cassette. (**b**) Detection of hCD2- *Tff2* mRNA in spleen and thymus of *CD2-Tff2* transgenic mice by RT- PCR analysis (top panel). WT (1, 2) and *CD2-Tff2* (3-6) mice samples are shown: splenic (1, 3, 5) and thymic mRNA (2, 4, 6). Negative controls, reactions performed without mRNA template (H_2O) are shown in lanes (7, 8). Middle panel shows negative controls, an amplification of above samples but without RT reaction. Bottom panel shows positive controls, a presence of *GAPDH* amplification products in the tested samples. (**c**) TFF2 peptide is detected in stomach, spleen and thymus of unchallenged *CD2-Tff2* transgenic mice (lower panel), β-actin (5 minutes of exposure, middle panel), β-actin (25 minutes of exposure, upper panel). (**d**) *CD2-Tff2* mice express a higher level TFF2 in spleen and thymus compared with wild-type counterparts. (**e-f)**, unchallenged *CD2-Tff2*, wild-type and *Tff2-null* mice (7-9 weeks old) do not differ in numbers of CD11b$^+$Gr-1$^+$ cells in spleen, blood, bone marrow (**e)** (*n*=5-9 mice per group, data present mean±s.d., one-way ANOVA test following Holm-Sidak's test, ns, non-significant) and in numbers of CD4$^+$, CD8$^+$ T-cells, B-cells, natural killers (NK) and dendritic cells (**f**) in spleen (*n*=3-9 mice in each group, one-way ANOVA test following Holm-Sidak's test, ns, non-significant). Data show the percentages of cell populations gated among single live CD45$^+$ cells for B-, dendritic cells and natural killers (NK). CD4$^+$ and CD8$^+$ cells are shown among single live CD3$^+$ population.

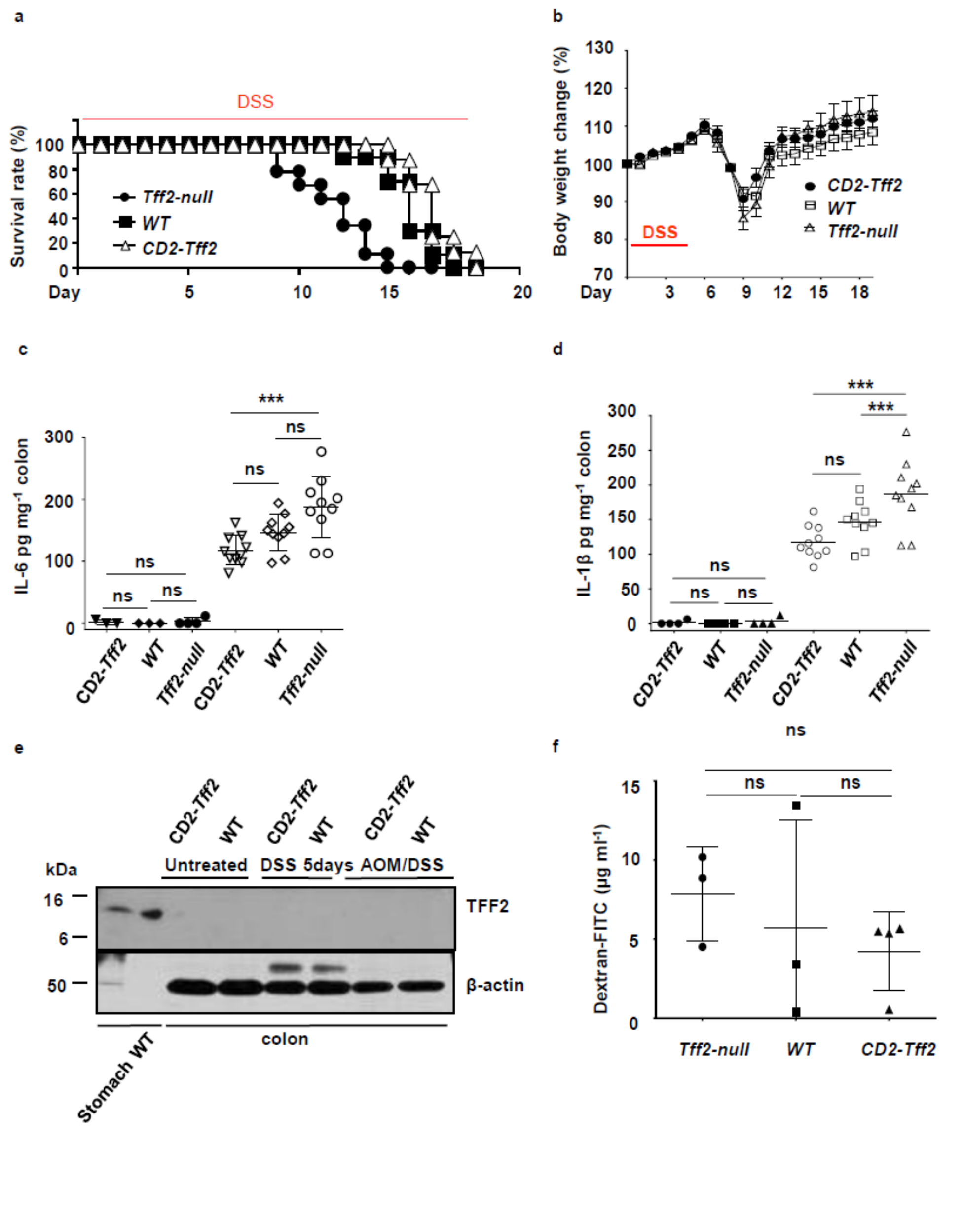

Supplementary Fig.3

Supplementary Figure 3. Susceptibility of WT, *CD2-Tff2* and *Tff2-null* mice to DSS treatment. a, Survival rate of *CD2-Tff2*, wild-type and *Tff2-null* mice upon continuous administration of 3% DSS (*n*=12 *TFF2-null*, *n*= 8 wild-type, *n*=7 *CD2-Tff2* mice). *TFF2-null* mice showed a highest mortality rate (*P*=0.00033, hazard ratio=8, Log-rank (Mantel Cox) test). (**b**) No difference in body weight loss between all groups of mice upon DSS 3% treatment during 5 days, n=4-5 mice in each group, ns, non-significant, **P*>0.5, one-way ANOVA test. (**c-d**). No difference in IL-6 (n=10 mice in each group) and IL-1β (n=10 mice in each group) level between *CD2-Tff2* and wild-type mice, ns, non-significant, *P>0.05 and ****P*<0.001, Kruskal-Wallis test after one-way ANOVA. (**e**) No TFF2 peptide was detected by western blot in colon from DSS and AOM/DSS-treated and untreated wild-type and *CD2-TFF2* mice. (**h**) colonic permeability does not differ between wild-type, *CD2-Tff2* and *Tff2-null* mice, two independent experiments, *n*=3-4 mice in each group: ns, non-significant, *P*>0.005, Kruskal-Wallis test after one-way ANOVA test.

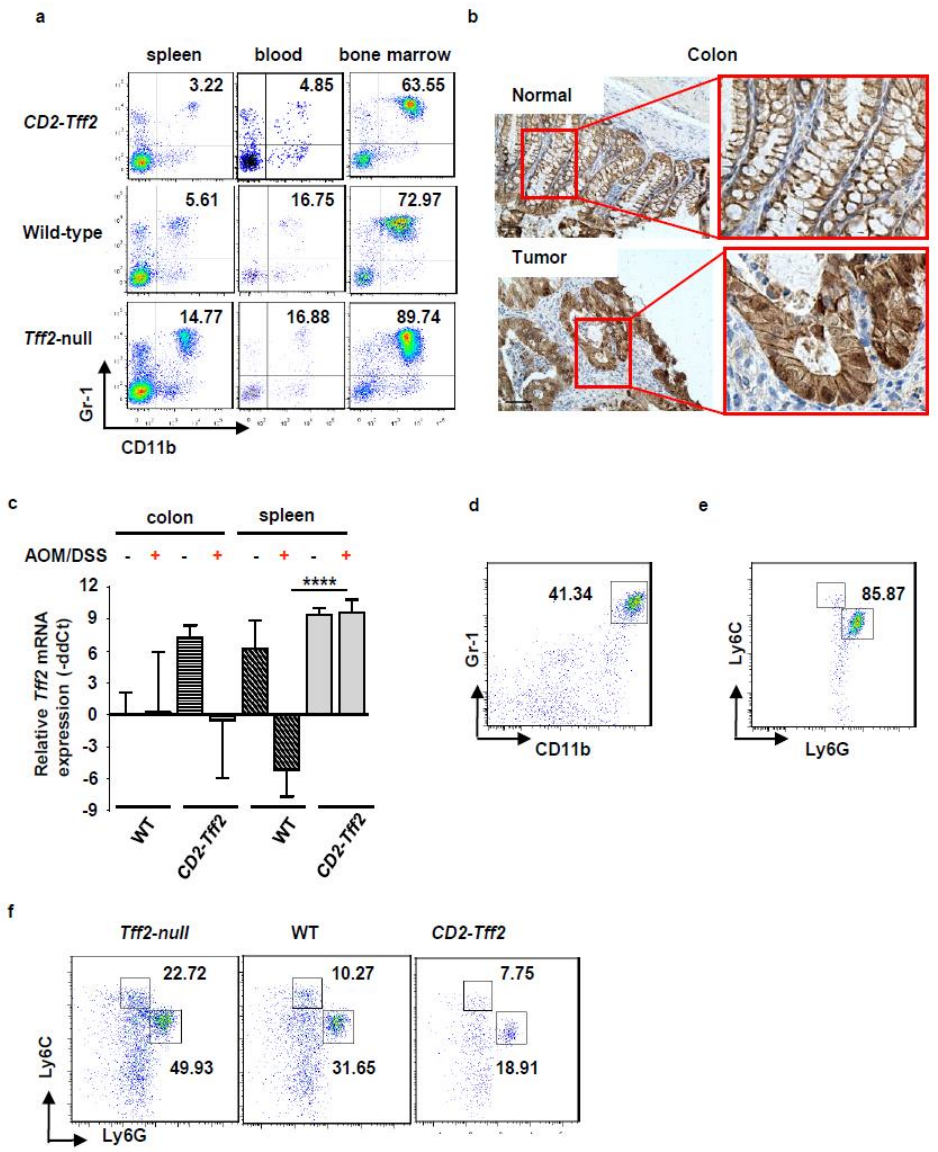

Supplementary Fig.4

Supplementary Figure 4. **Accumulation of CD11b$^+$Gr-1$^+$ cells in tumor-bearing mice.** (**a**) Representative flow cytometry dot plots showing the percentage of CD11b$^+$Gr-1$^+$ cells in spleen, bone marrow and blood from *Tff2*-null, wild-type and *CD2-Tff2* mice treated AOM/DSS and sacrificed 5 months later. CD11b$^+$Gr-1$^+$ cells were evaluated as percentage of single live cells. Representative data from 4 independent experiments, n=3-6 mice in each group (**b**) Activation of β-catenin: nucleus localization of β-catenin in tumor vs. healthy tissues of colon from tumor-bearing *Tff2-null* mouse. Scale bar is 50 µm. (**c**) *TFF2* mRNA is high in the spleens of *CD2-Tff2* mice but decreases in the spleens of WT animals and the colonic tissues of WT and *CD2-Tff2* mice in AOM/DSS model. Data collected from 4-5 mice in each group, data are the mean ± s.e.m. of triplicates (Student's t-test, **** $P<0.0001$). (**d**) CD11b$^+$Gr-1$^+$ cells in colon tumor are shown among single live CD45$^+$ cells. (**e-f**) Preponderance of Ly6G$^+$ over Ly6C$^+$ subset in colon tumor (**e**) and spleen (**f**) in AOM/DSS model. CD11b$^+$Ly6C$^+$ and CD11b$^+$Ly6G$^+$ cells were gated among single live CD45$^+$ cells. Data collected from 5 mice.

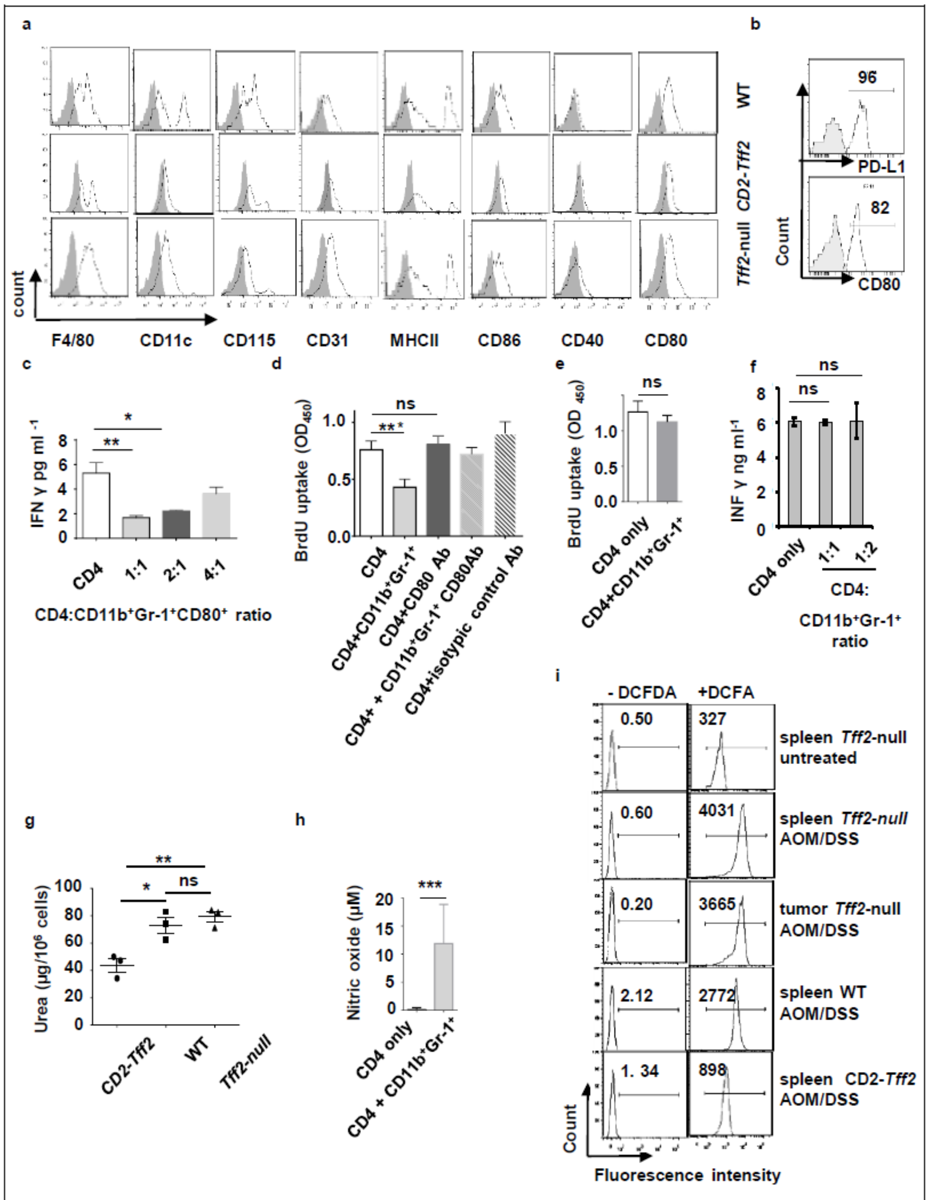

Supplementary Fig. 5

Supplementary Figure 5. **Phenotypic and functional characterization of $CD11b^+Gr\text{-}1^+$ cells from mice treated with AOM/DSS.** (**a**) Phenotype of splenic $CD11b^+Gr\text{-}1^+$ cells in mice at 5 months age after AOM/DSS treatment. Note that CD80 antigen is highly expressed by $CD11b^+Gr\text{-}1^+$ splenic cells from tumor-bearing *Tff2-null* and wild-type mice in contrast to *CD2-TFF2* transgenic mice. Cells were gated on single viable $CD11b^+Gr\text{-}1^+$ population, and expression of respective markers was analyzed. Isotype control is shown as shaded and respective marker as unshaded histogram. (**b**) $CD11b^+Gr\text{-}1^+$ cells from tumor of *Tff2-null* mice express PD-L1 and CD80 antigens from moderate to high. (**c**) $CD11b^+Gr\text{-}1^+$ cells from *Tff2-null* and wild-type mice suppress INF-γ production by $CD4^+$ T-cells stimulated with anti-CD3- and CD28- bound antibody. Data are mean ± s.e.m. of triplicates, * $P<0.05$; ** $P<0.01$, unpaired t-test, two-tailed. (**d**) CD80 Ab abrogates suppressive effect of $CD11b^+Gr\text{-}1^+CD80^+$ cells. Assay was performed in triplicate and data are mean± s.e.m. Representative data from two experiments, ** $P<0.01$, unpaired t-test, two-tailed. (**e**, **f**) $CD11b^+Gr\text{-}1^+$ cells from *CD2-Tff2* do not suppress $CD4^+$ T cells proliferation and INF-γ production, assay was performed in triplicate, and data are mean±s.e.m. of replicates, *$P>0.05$, ns, non-significant, unpaired *t*-test was used to determine significance. **g**, Arginase activity in the $CD11b^+Gr\text{-}1^+$ from spleens of AOM/DSS treated *CD2-Tff2*, wild-type and *Tff2-null* mice (n=3 in each group). Assay was performed in triplicate; data d express μg of urea per sorted 10^6 cells. * $P<0.05$, ** $P<0.01$, ns, non-significant, Holm-Sidak's multiple comparison after Anova test. (**h**) Nitric oxide activity in splenic $CD11b^+Gr\text{-}1^+$ cells from tumor-bearing mice. Assay was performed at triplicate and data are mean ± s.e.m., *$P<0.05$ unpaired two-tailored test. (**i**) $CD11b^+Gr\text{-}1^+$ from spleen of tumor-bearing mice and colon tumor express high ROS activity. Data pulled out of three mice from each group, numbers represent Geometric mean to measure the shift in fluorescent intensity.

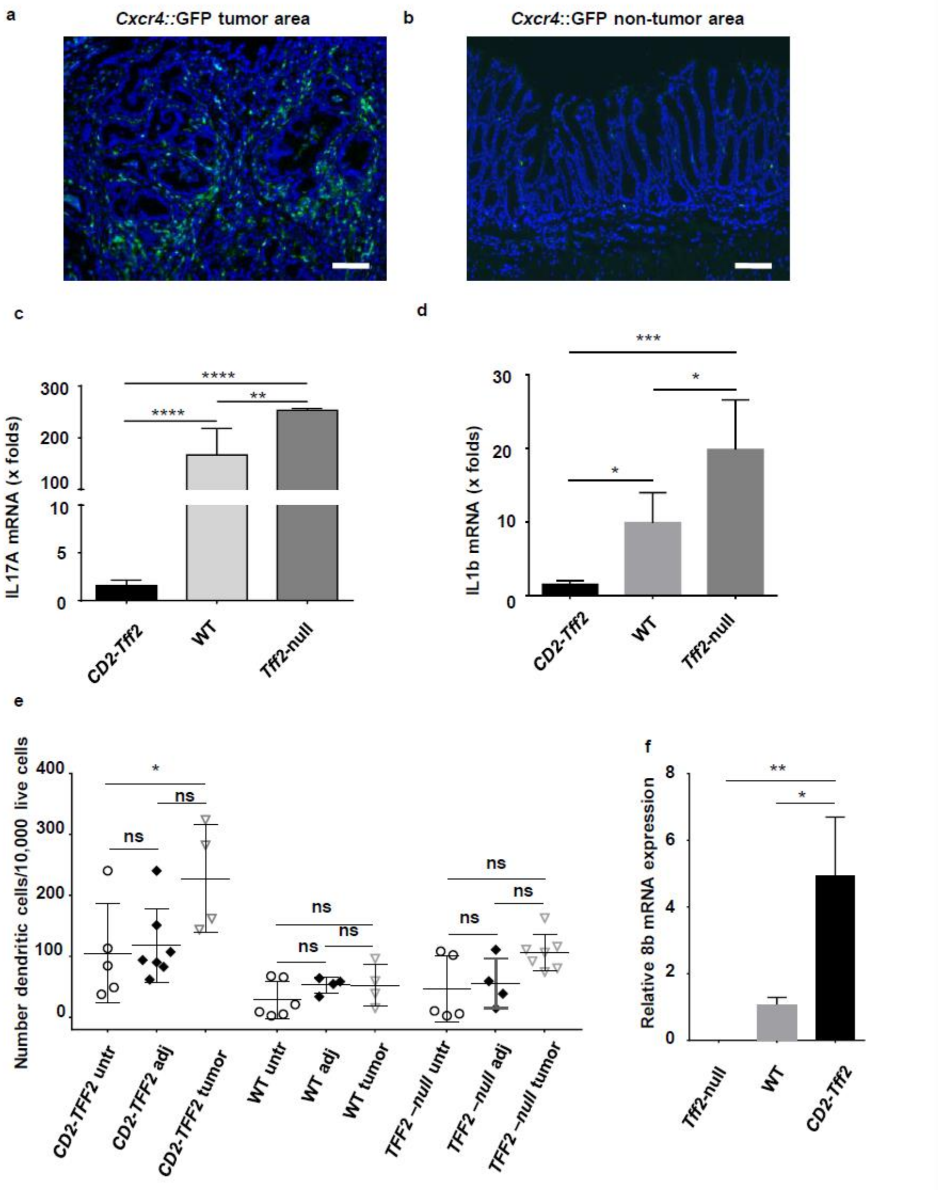

Supplementary Fig. 6

Supplementary Figure 6. Characterization of colon tissues from AOM/DSS treated mice. (**a-b**) higher accumulation of CXCR4-GFP cells in the colon tumor area (**a**) vs. non-tumor tissues (**b**). *Cxcr4-GFP* mice are treated with AOM/DSS, and sacrificed at 20 weeks. Scale bar is 100 μm. (**c-d**) high level of pro-tumorigenic cytokines IL17A and IL-1â in tumor of wild-type and *Tff2-null* compare with *CD2-Tff2* mice, data are mean±s.d. pulled out from 3-5 mice in each group, **P*<0.05, ****P*<0.001, *****P*<0.0001, Holm-Sidak's multiple comparison test after one-way ANOVA test. Total mRNA was isolated from tumor colonic tissues and transcripts of IL17A (b) and IL-1a (c) were analyzed by real-time PCR in triplicates. (**e**) *CD2-Tff2* mice have higher numbers of dendritic cells in tumor compare with tumor in wild-type and *Tff2-null* mice, each dot represent one mouse, n=4-7 mice in each group, ***P*<0.05, Sidak's multiple comparison test after one-way ANOVA test. (**f**) The *Tff2-null* mice with the greatest numbers of MDSCs had undetectable colonic $CD8^+$ T cells. mRNA was isolated from paraffin sections of colon samples obtained from AOM/DSS- treated mice 6 months later (3-4 mice from each group). Data shown are the mean ± s.e.m. of triplicate determinations, **P*<0.05.

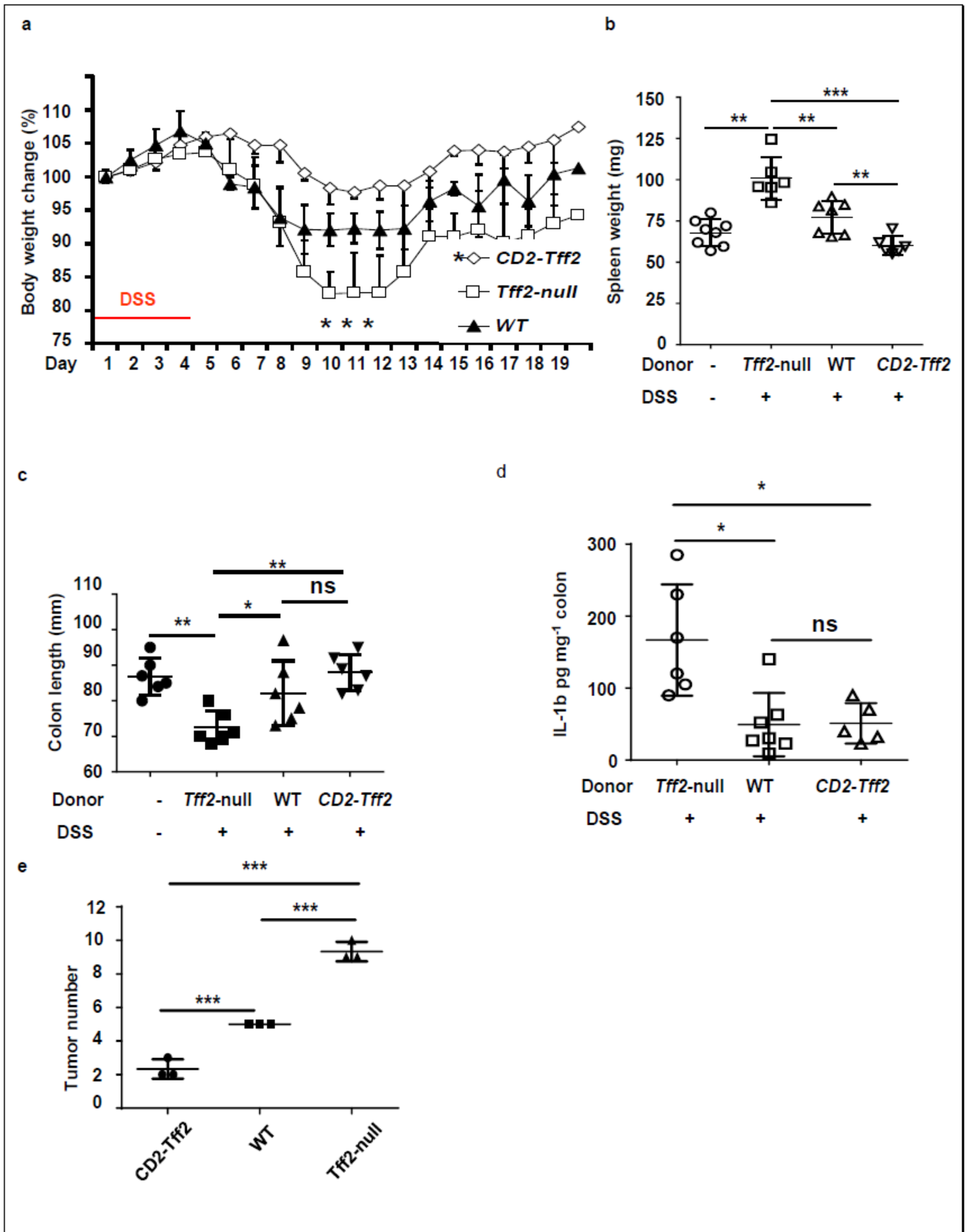

Supplementary Fig.7

Supplementary Figure 7. **TFF2 expressed in lymphohematopoietic compartment decreases systemic and colonic inflammation (DSS model) and tumor burden as well.** (**a-d**) Wild-type mice were transplanted with the bone marrow from wild-type, *Tff2-null*$^{-}$ and *CD2-Tff2* animals, subjected DSS protocol and analyzed on day 19. Note a highest weight loss (**a**), splenomegaly (**b**), colon shrinking (**c**) and increased level of IL-1β (**d**) in chimaeras with bone marrow transplanted from *Tff2-null* while chimaeras with bone marrow from *CD2-Tff2* mice showed lowest body weight and normal spleen mass (6-7 mice in each group; ns, non-significant, * *P*<0.05, ***P*<0.01, ***P<0.001, Sidak's multiple comparison test after ordinary one-way Anova test). Representative data one from two experiments. (**e**) Wild-type mice with bone marrow transplanted from *Tff2-null* mice develop more tumors versus chimaeras with bone marrow from wild-type or *CD2-Tff2* transgenic mice in AOM/DSS model (*n*=3 per group, ANOVA test followed Sidak's-multiple comparison test, ***, *P*<0.001).

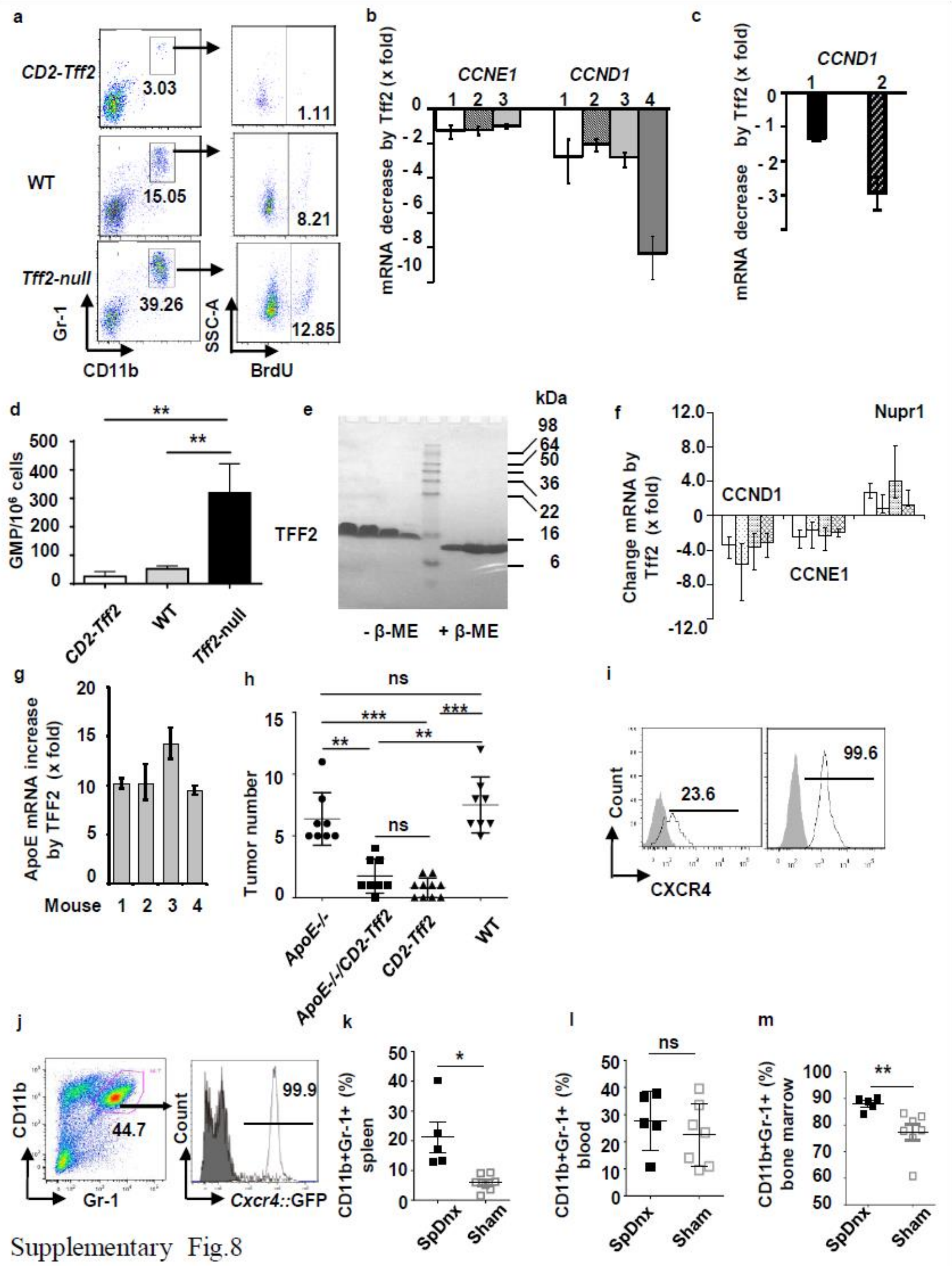

Supplementary Fig.8

Supplementary Figure 8. TFF2 is directly responsible for the expansion of IMC/MDSCs. (**a**) Splenic CD11b$^+$Gr-1$^+$ cells from *Tff2*-null mice show higher BrdU incorporation vs. IMCs from wild-type and *CD2-Tff2* mice**.** (**b, c**) rTFF2 down-regulates cyclin D1 (*CCND1*) along with cyclin E1 (*CCNE1*) in MDSC and in IMC *in vitro*. CD11b$^+$Gr-1$^+$ cells were sorted from spleens of tumor-bearing mice and cultured in presence of rTFF2 (4 mice) (**b**) or DSS–treated mice (2 mice), each bar represents mean± s.e.m of triplicates from one mouse. (**d**) Increased number of GMP in spleen of wild-type and *Tff2*-null mice treated with DSS. Data collected from 3-4 mice in each group and present mean±s.d.. (**e**) Purified mouse rTFF2 is homogenous under nonreducing/reducing conditions. (**f-g**) Validation of microarray data for cyclin D1 (*CCND1*), cyclin E1 (*CCNE1*) down-regulation and up-regulation of *Nupr1* (**f**) and apolipoprotein E (*ApoE*) (**g**) by qRT-PCR. Data pulled out from 4 mice, each bar represents mean± s.e.m of triplicates from one mouse. **h**, *ApoE* loss did not reverse *TFF2* phenotype. *ApoE-/-*, *CD2-Tff2*, *ApoE-/-/CD2-Tff2* and WT mice were subjected standard AOM/DSS protocol and analyzed 5 months later. Combined data from two independent experiments, n=7-10 in each group, ns, nonsignificant, $^{**}P<0.01$, ***, $P<0.001$, one-way ANOVA followed Holm-Sidak test (**i)** Expression of CXCR4 on CD11b$^+$Gr-1$^+$ cells in spleen of untreated (left panel) and in tumor-bearing *Tff2-null* (right panel) and tumor CD11b$^+$Gr-1$^+$ from *Cxcr4-GFP* mice (**j)** Cells were gated on single viable CD45$^+$CD11b$^+$Gr-1$^+$ cells, shaded histogram represents isotypic control, unshaded histogram represents CXCR4 expression. (**k-m**) proportion of MDSC in spleen (**k**), blood (**l**) and bone marrow (**m**) of wild-type mice with splenic denervation (SpDnx) and Sham mice. n=5-7 mice in each group; ns, non-significant, $^{*}P<0.05$, $^{**}P<0.01$, unpaired t-test two-tailed.

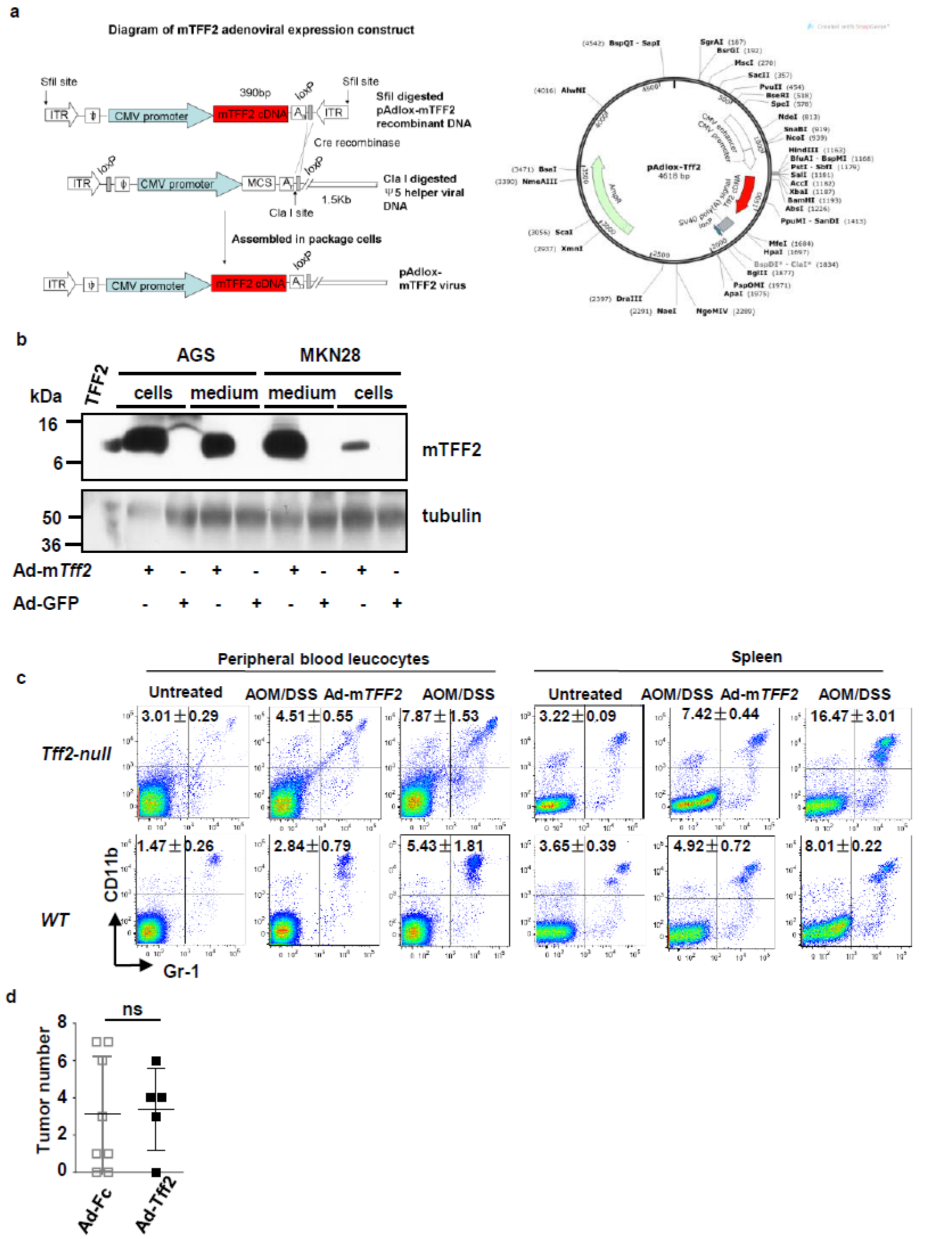

Supplementary Fig. 9

Supplementary Figure 9. Adenovirus delivery of TFF2 suppresses colon tumorigenesis. (**a**) Diagram of mTFF2 adenoviral expression construct (left panel- diagram scheme of cloning, right panel- diagram of plasmid). (**b**) Secretion of TFF2 in the medium from cancer cell lines transfected with Ad-m*Tff2*. (**c**), representative data of FACS analysis of splenic, bone marrow and circulating MDSC in wild-type and *Tff2*-null mice treated with Ad-m*Tff2* versus control Ad-Fc. (**d**) TFF2 adenovirus delivery does not rescue *Rag2*$^{-/-}$ mice from AOM/DSS-induced tumorigenesis. N=5-8 mice in group, *P<0.05, unpaired t-test, two-tailed.

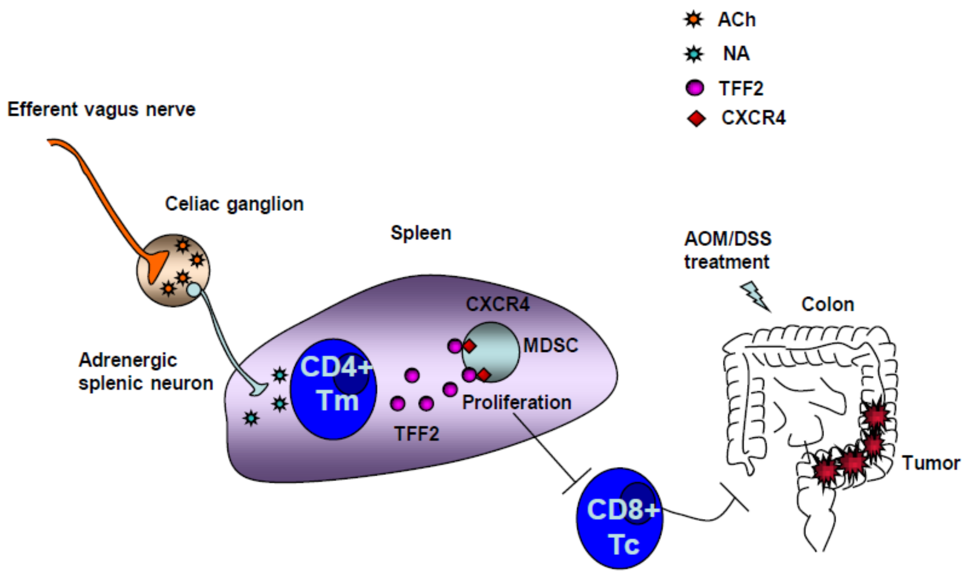

Supplementary Fig. 10

Supplementary Figure 10. **Scheme of TFF2 action through the vagal anti-inflammatory reflex.** An impulse from the vagus nerve activates an adrenergic fiber within the celiac ganglion. In turn this releases noradenaline to activate memory T cells within the spleen. These T cells releases TFF2 that causes downregulation of cyclin D1 on IMCs/MDSCs via CXCR4. This inhibits their expansion releasing cytotoxic CD8 T cells from their suppression and allowing $CD8^+$ T cells to suppress colonic carcinogenesis.

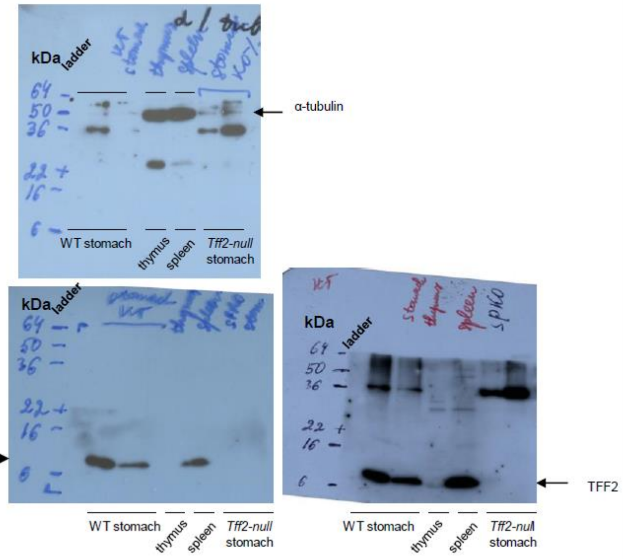

Supplementary information to Figure 1a.

Identification of TFF2 peptide in normal spleen of wild-type mice by western blot.

Supplementary figure 11

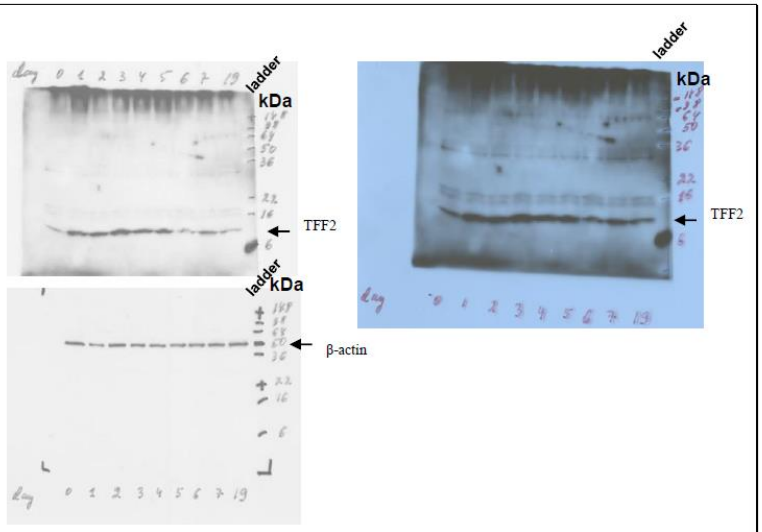

Time course of expression in spleen TFF2

Supplementary information to Figure 1b

Time-course change of the TFF2 protein

Supplementary figure 11

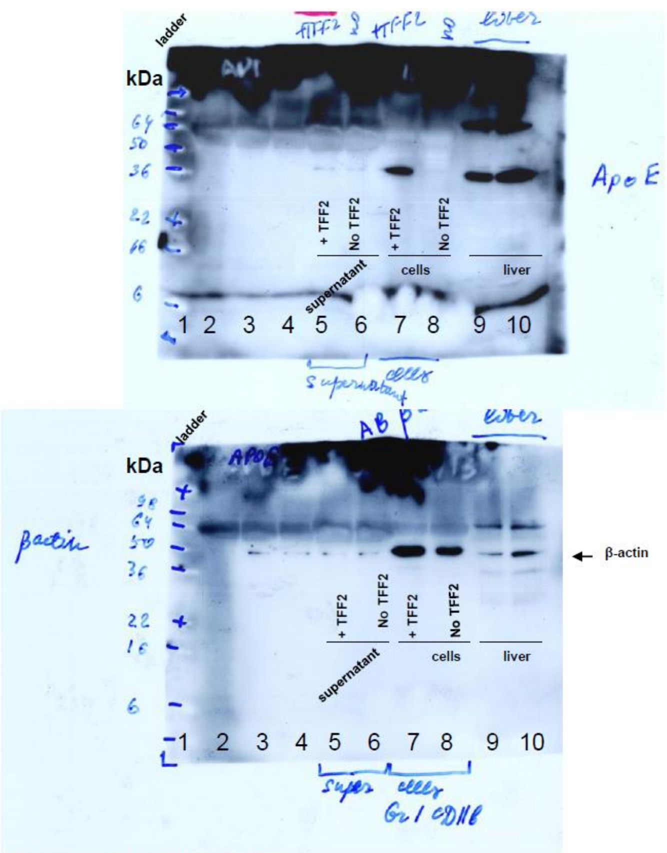

Supplementary information to Figure 4e

rTFF2 induced expression of membrane-bound ApoE in CD11b+Gr1+ cells.

Supplementary figure 11

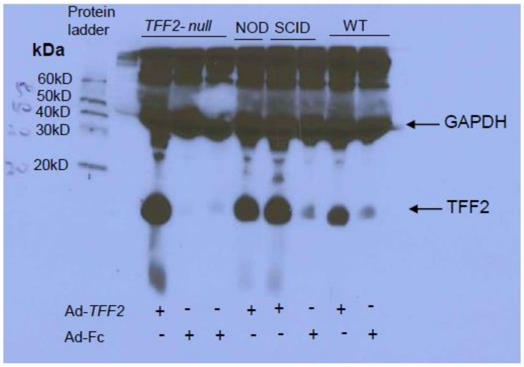

Supplementary information to Figure 5a.

Detection of recombinant TFF2 by western blot in serum from *Tff2-null*, NOD, SCID and WT mice 5 days after Ad-m*Tff2* treatment (5×10^8 pfu per mouse).

Supplementary figure 11

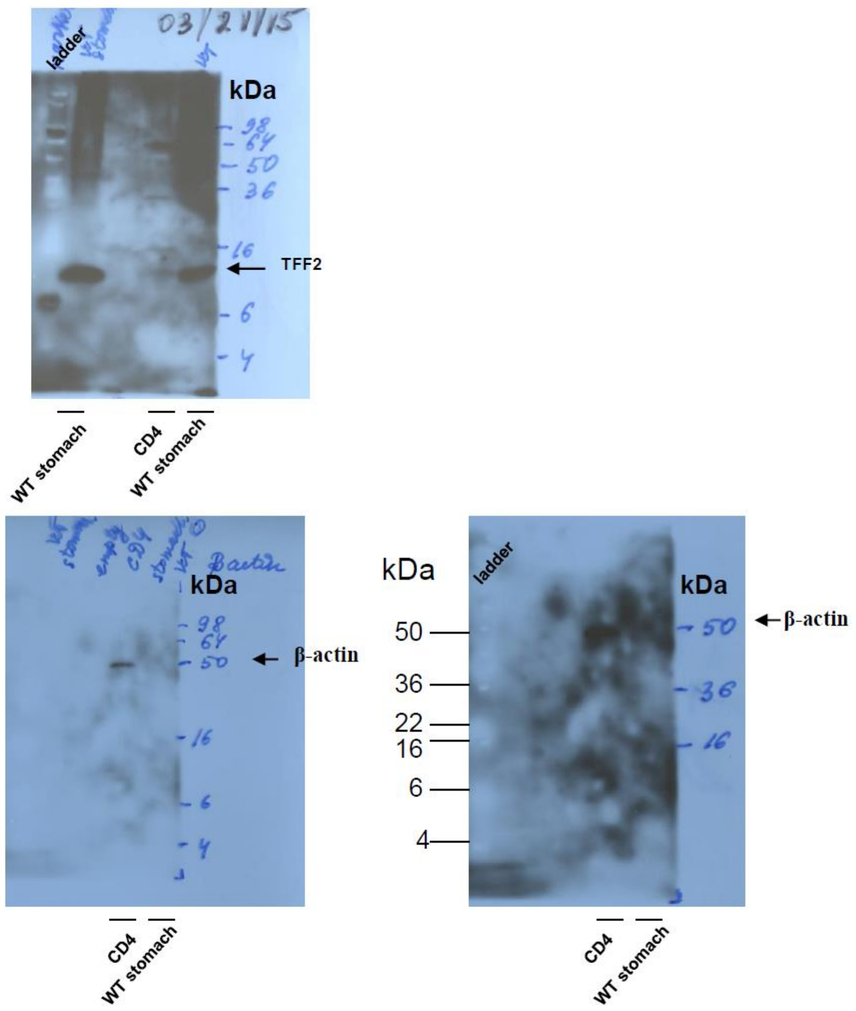

Supplementary information to Supplementary Figure 1d.

Western blot analysis of TFF2 expression in splenic CD4+ T cells. CD4 T cells were isolated from DSS-treated mice (day 3) and stimulated with cell stimulation cocktail with bleferdin A.

Supplementary figure 11

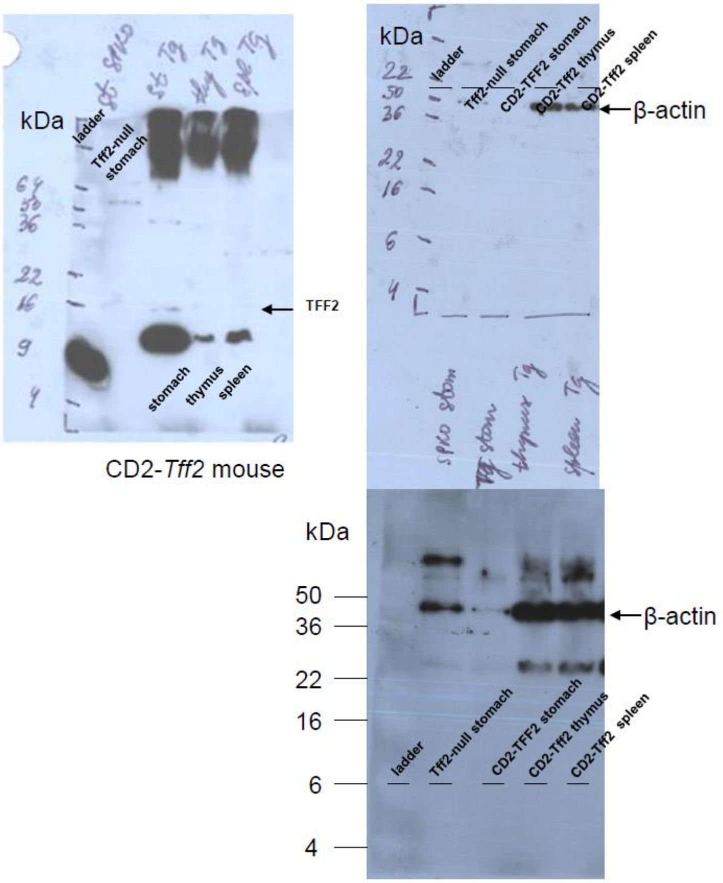

Supplementary information to Supplementary Figure 2c.

TFF2 peptide is detected in stomach, spleen and thymus of unchallenged *CD2-Tff2* transgenic mice

Supplementary figure 11

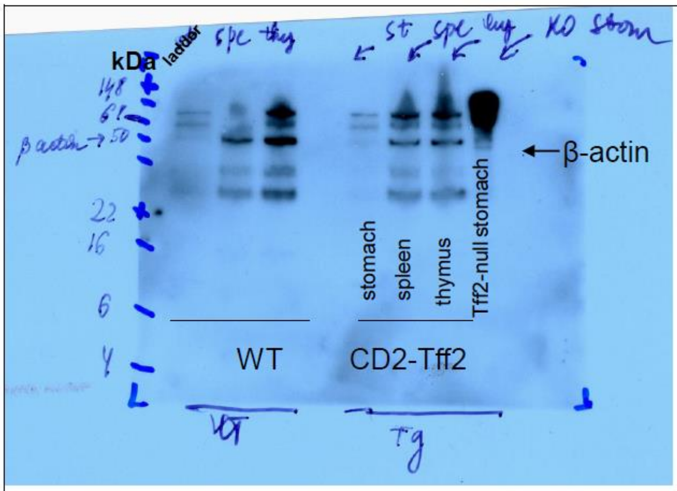

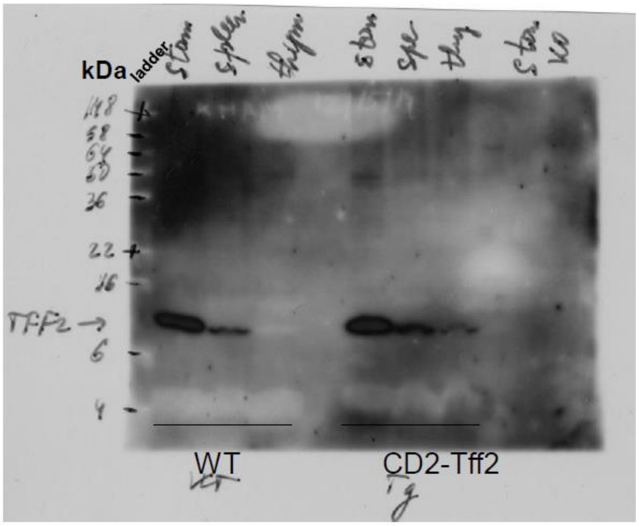

Supplementary information to Supplementary Figure 2d

CD2-Tff2 mice express a higher level TFF2 in spleen and thymus compared with wild-type counterparts.

Supplementary figure 11

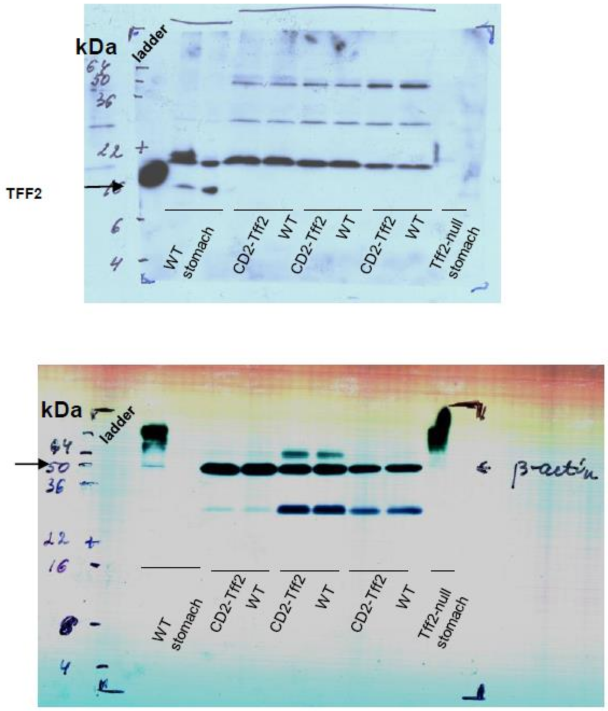

Supplementary information to Supplementary Figure 3e

No TFF2 peptide was detected by western blot in colon from DSS and AOM/DSS-treated and untreated wild-type and *CD2-TFF2* mice.

Supplementary figure 11

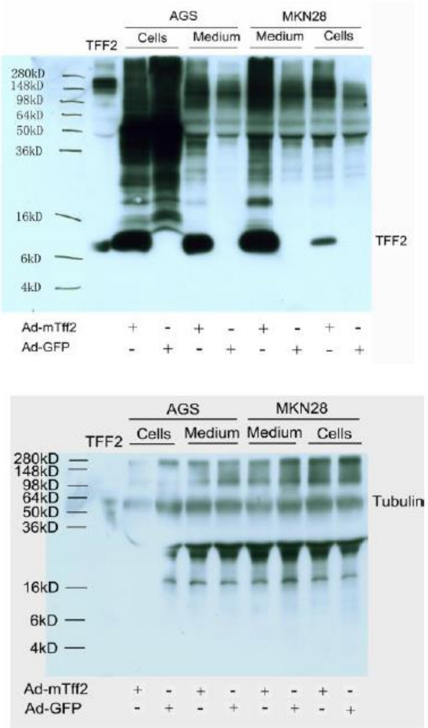

Supplementary information to Supplementary Figure 9b.
Secretion of TFF2 in the medium from cancer cell lines transfected with Ad-m*Tff2*.

Supplementary figure 11

Supplementary Table 1. Characterization of *CD2-Tff2* mice. Blood count.

Mice / Cell types	CD2-Tff2	Wild-type	P* value
**Basophils**	5.59±7.23	2.09±1.13	0.9365
**Eosinophils**	14.32±6.11	27.52±18.4	0.5317
**Segmented**	592.95±319.81	667.61±12.23	0.4127
**Lymphocytes**	2024.3±815.17	3050.10±695.55	0.0556
**Monocytes**	130.99±47.77	196.014±58.40	0.0952
**WBC ($x10^3$/ul)**	2.79±1.14	3.94±0.85	0.2424
**RBC ($x10^6$/ul)**	10.3±0.67	9.24±0.26	0.0519
**platelets**	617.6±175.11	576.5±78.55	0.6494
**RDW (%)**	13.94±1.47	13.34±0.93	0.3896
**Reticulocytes (%)**	0	0.006±0.013	0.3466
**Hct (%)**	50.93±4.46	47.77±1.42	0.2581
**Hgb(g/dL)**	13.35±1.26	13.73±0.4263	13.73±0.4263

*, Mann-Whitney test, two-tailed

Supplementary Table 2. Body and spleen weight of unchallenged wild-type and *CD2-Tff2* mice

**Phenotype** / **Gender**		***CD2-Tff2***	**Wild type**	**P* value**
**Male weight**	**Body (g)**	22.6±1.04 (*n*=9)	22.7±1.1 (*n*=9)	0.9999
	**Spleen (mg)**	64.45±8.7 (*n*=6)	67.75±5.8 (*n*=6)	0.6753
**Female weight**	**Body (g)**	18.7±1.4 (*n*=9)	19.0± 0.8 (*n*=7)	0.6517
	**Spleen (mg)**	70.6±5.6(*n*=5)	66.9±7.9(*n*=6)	0.8095

*, Mann-Whitney test, two-tailed

Supplementary Table 3. Selected genes regulated by TFF2 in CD11b+Gr-1+ cells

**Symbol**	**Description**	**Fold Change**
**Apoe**	apolipoprotein E	89.8
**Mcpt8**	mast cell protease 8	72.5
**Cxcl5**	chemokine (C-X-C motif) ligand 5	62.8
**Cpa3**	carboxypeptidase A3, mast cell	54.7
**Il12a**	interleukin 12a	33.1
**F2r**	coagulation factor II (thrombin) receptor	30.8
**Ctsg**	cathepsin G	20.9
**Fcer1a**	Fc receptor, IgE, high affinity I, alpha polypeptide	19.9
**Ly86**	lymphocyte antigen 86	-19.7
**Klrb1b**	killer cell lectin-like receptor subfamily B member 1B	-19.3
**CcnD1**	Cyclin D1	-8.3
**CcnE 1**	Cyclin E1	-2.29
**Nupr1**	Nuclear protein 1	2.68
